# Simulating COVID-19 lockdowns’ impact on depressive symptoms in the northern Netherlands

**DOI:** 10.1186/s12889-026-27001-3

**Published:** 2026-03-21

**Authors:** Kristina Thompson, Jiri Kaan, Ji Qi, Astrid Anh Pham, Eva Viviani

**Affiliations:** 1 Health & Society, Wageningen University & Research, Hollandseweg 1, Wageningen, 6706 KN The Netherlands; 2 Netherlands eScience Center, Science Park 402, Amsterdam, 1098 XH The Netherlands

**Keywords:** COVID-19, Lockdowns, Mental health, Depressive symptoms, Microsimulation

## Abstract

**Background:**

During the COVID-19 (SARS-CoV-2) pandemic, a major challenge for policymakers was finding the balance between containing outbreaks and supporting mental health. Although lockdowns slowed COVID-19 transmission, it appears that they also increased the prevalence of depressive symptoms. It is not yet clear whether different, hypothetical lockdown scenarios would have differently impacted the prevalence of depressive symptoms.

**Methods:**

To that end, we developed an open-source microsimulation model, COMMA (COvid Mental health Model with Agents). With COMMA, we explored how lock down scenarios, individual characteristics, and behaviors during lockdowns related to the prevalence of depressive symptoms during the COVID-19 period. The characteristics of the population were synthesized from Lifelines, a prospective cohort study set in the northern Netherlands.

**Results:**

COMMA simulations estimated that the actual lockdown scenario between June 2020 and June 2021 would result in 10.92% (95% CI: 10.68%-11.18%) of the population experiencing depressive symptoms, up from 3.49% (95% CI: 3.49–3.49) at baseline. Had there been a full or partial lockdown for the entirety of this period, 11.44% (95% CI: 11.18%-11.69%), or 10.63% (95% CI: 10.38%-10.87%), of the population, respectively, would have experienced depressive symptoms.

**Conclusions:**

Lockdown severity was an important predictor of the prevalence of depressive symptoms, with higher rates in full lockdowns than partial lockdowns. However, across all simulations, only a minority of the population ever developed depressive symptoms. These findings suggest that lockdowns, particularly full lockdowns, are not without consequences for mental health.

**Supplementary Information:**

The online version contains supplementary material available at 10.1186/s12889-026-27001-3.

## Background

The SARS-CoV-2 (COVID-19) pandemic provides important lessons for preparedness for future pandemics. For instance, reflecting on this period may help to identify the appropriate balance between containing outbreaks and supporting population mental health and well-being. Although non-pharmaceutical interventions helped to slow the spread of COVID-19, they were not without negative consequences of their own. This was particularly the case for lockdowns, which included measures such as isolating at home, restrictions on social gatherings, restrictions on travel, and restrictions on movement, and, in some cases, curfews [1].

In particular, sub-clinical measures of depressive symptoms, which may be more sensitive to shorter-term changes in mental health and ultimately lead to depressive disorders, seemed to experience especially sharp increases in prevalence [2, 3]. Prior to the COVID-19 pandemic, global estimates of the prevalence of depressive symptoms ranged between 4% and 13% [4, 5]. During the pandemic, there is evidence that nearly one in five adults experienced depressive symptoms [6]. Understanding the consequences of lockdowns on depressive symptoms is therefore crucial to fully identify the trade-offs for population health that lockdowns may have.

Although a number of studies have examined lockdowns’ relationship to depressive symptoms, the vast majority of these studies explored this relationship empirically. This limits our knowledge on the topic to what was actually observed. To more fully understand the mental health trade-offs of lockdowns, exploring “what if” hypothetical policy scenarios is important. Employing simulation is a cost-effective and valid way of doing so [7]. During the height of the COVID-19 pandemic, simulation was frequently used to estimate the spread of COVID-19, and to identify the economic and epidemiological trade-offs of different lockdown policies [8–10]. However, simulations exploring the impact of lockdowns on mental health remain scarce.

An exception to this is Romanyukha et al. [11], in which the authors simulated the trade-off between quality of life years gained due to lockdowns, and life years lost due to lockdown-related mental health disorders in Moscow with an agent-based simulation of Susceptible-Infectious-Recovered dynamics (ABM-SIR). While this model has a number of strengths, it estimates its outcome, increased stress, largely as a function of mobility, with stress load increasing as mobility decreases [11]. While there is evidence of a moderate relationship between decreased mobility and decreased stress [12], this likely understates the complex interplay of determinants of mental health. For instance, there is evidence that individuals during the COVID-19 pandemic were able to buffer stressful aspects of lockdowns by engaging in positive behaviors, or worsen lockdowns’ effects by engaging in negative coping behaviors [13–15]. In the present study, we sought to more fully capture a wider variety of determinants of mental health-related actions and depressive symptoms.

To that end, we developed a microsimulation model simulating lockdowns’ relationship to depressive symptoms. Microsimulation models are particularly well-suited to this purpose. Microsimulation incorporates individuals’ different characteristics at baseline, and incorporate these different characteristics into individuals’ trajectories [16]. Unlike in many other modelling types, individuals in a microsimulation represent unique individuals, rather than averages from a given population [17]. They also are frequently used to study the impact of macro-level events, such as the implementation of health policies [17]. These features make microsimulation a powerful tool for studying macro-level events on individuals’ mental health, although they remain under-used in psychiatric epidemiology [17].

We set our model in the Netherlands. Relative to other European countries, the Netherlands implemented a wider range of lockdown measures, and over a longer period of time. In the first three months of the pandemic, the Dutch government implemented a so-called “intelligent lockdown,” with fewer and less severe restrictions on movements than in neighboring countries such as Belgium and Germany [18]. However, as many European countries more fully opened up over the course of 2021, the Netherlands entered a full lockdown with curfew restrictions in autumn 2021 until February 2022 [19]. The Netherlands therefore is an informative context in which to simulate lockdowns’ relationship to depressive symptoms. Against this backdrop, we examined various lockdown scenarios’ relationship to the development of depressive symptoms.

## Methods

To do so, a microsimulation model, COMMA (COvid-19 Mental health Model with Agents) was developed. COMMA is open-source and available via: https://github.com/covid19ABM/comma.

### Data sources

COMMA’s synthetic population was derived from the Lifelines Biobank and Cohort Study (Lifelines), a multidisciplinary prospective population-based cohort study set in three northern Dutch provinces, Groningen, Friesland and Drenthe [20, 21]. Adult participants were asked to complete several self-administered questionnaires on topics including demographics, socioeconomic status, and lifestyle behaviors. Before entering the study, each participant signed an informed consent form. The Lifelines study was conducted according to the principles of the Declaration of Helsinki and approved by the Medical Ethics Committee of the Institutional Review Board of the University Medical Center Groningen, the Netherlands (2007/152). A detailed description of the Lifelines cohort study can be found elsewhere [20, 22].

Lifelines provides rich information on participants prior to the COVID-19 pandemic, from which we derived baseline characteristics of agents, or synthetic individuals representing Lifelines participants. We took this from the second wave of Lifelines’ assessments, taking place between January 2014 and December 2017, with a median of September 2015. This was the most recent wave prior to the COVID-19 pandemic.

Between March 2020 and October 2022, Lifelines participants were asked to take part in a special COVID-19 study, to monitor changes in behaviors and health outcomes during the pandemic [23]. The questionnaires issued as part of this study changed in frequency over the course of the pandemic. Between 30 March and 29 May 2020, weekly questionnaires were issued. Thereafter, questionnaires varied in frequency between fortnightly and monthly. From this COVID-19 dataset, we derived agents’ actions, or behaviors, and likelihood of developing depressive symptoms during the pandemic [24]. Different questions were also asked in different questionnaires.

The Lifelines sample, from which we generated COMMA’s synthetic population, was selected in several steps. First, participants had to have taken part in the second wave of Lifelines’ main assessments (*n* = 101,751). This was the last wave completed prior to the COVID-19 pandemic. Second, participants had to have taken part in at least one of the COVID-19 Lifelines study (*n* = 61,778). Third, participants had to be between the ages of 24 and 65 in 2020, so they would have been working age during the COVID-19 pandemic. This yielded a sample of 52,378 participants on which this study’s synthetic population is based.

To capture (1) baseline characteristics’ influence on different actions taken during the pandemic, and (2) different actions’ likelihood of depressive symptoms during the pandemic, information from the COVID-19 questionnaires were used. We measured both of these relationships during two different lockdown types: either full or partial, as defined by the Dutch government [25]. While configuration of measures varied per lockdown, key features defined full and partial lockdowns. Full lockdowns always involved the closure of non-essential shops and orders to work from home for all non-essential workers. Generally, schools were closed or implemented distance learning. In partial lockdowns, bars and restaurants were closed, there were often limits on the number of people who could attend social gatherings, and non-essential workers were encouraged to work from home.

To derive values for (1) baseline characteristics’ influence on different actions taken during the pandemic, and (2) different actions’ likelihood of depressive symptoms during the pandemic, we used specific questionnaires representing partial or full lockdowns. Questionnaires were selected based on having the largest number of relevant questions asked. To measure full lockdowns, information from questionnaire 17 (5 January 2021 to 8 February 2021) and 18 (28 February 2021 to 25 March 2021) were used. To measure partial lockdowns, information from questionnaires 20 (26 April 2021 to 20 May 2021) and questionnaire 21 (25 May 2021 to 18 June 2021). Other questionnaires [5, 7, 9, 16, 24] were also used, and yielded similar results.

Information on COVID-19 cases was also used. Municipal daily case information was used. This information was provided by the Netherlands’ National Institute for Public health and the Environment (RIVM) [26].

### Variable selection and conceptual model

We first developed a conceptual model illustrating how and why individuals’ features at baseline and actions during the COVID-19 pandemic may have influenced the probability of having depressive symptoms, and, particularly, how these features and actions may differently impact the likelihood of developing depressive symptoms during different lockdown types (partial or full). We hypothesized that having a given set of characteristics was related to the probability of taking certain actions during lockdowns. In turn, the actions an individual might take during the COVID-19 pandemic would be related to their likelihood of having depressive symptoms.

We selected baseline features and actions during COVID-19 based on a review of the literature, highlighting factors relating to the development of depressive symptoms generally and in the context of the COVID-19 pandemic [27, 28]. Each of the selected individual characteristics have been shown to be related to at least one of the actions during lockdown. For instance, having attained a higher educational status was related to a higher probability of working from home [29], exercising [30], and seeking help from social networks [31] during the height of the COVID-19 pandemic. In turn, these actions are associated with higher (in the case of working from home) and lower (in the case of exercising and seeking help from social networks) probabilities of having depressive symptoms [32–35].

**Fig. 1:**
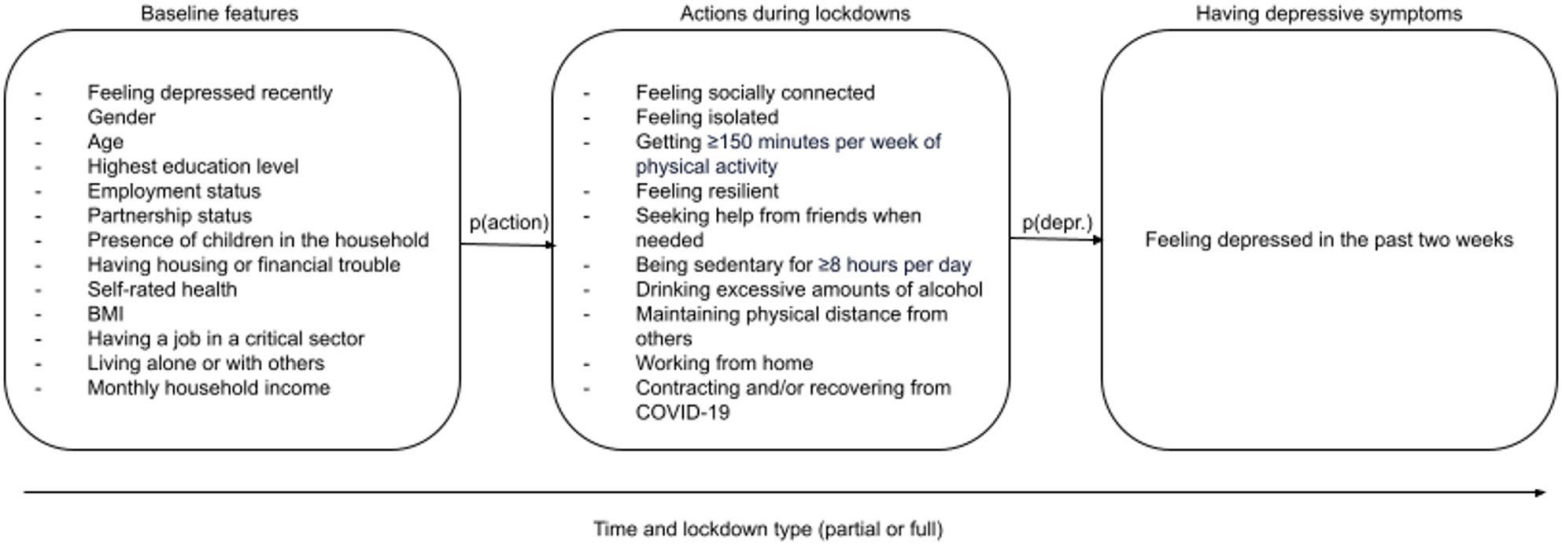
COMMA’s conceptual model

Figure 1 illustrates all baseline features and actions during lockdowns. A full list of the variables used to derive all baseline features and actions is included in Tables A.1 and A.2. Perhaps most importantly, we used the validated 8-item Mini International Neuropsychiatric Interview (MINI), asked both at baseline and in the COVID-19 questionnaires, to identify whether or not an individual experienced depressive symptoms [36]. We were explicitly interested in whether individuals experienced sub-clinical depressive symptoms, which are more prone to short-term fluctuations, and are important early warning signs of clinical depression [37]. In line with Qi et al. [38], which also used Lifelines COVID-19 questionnaire, we operationalized ‘having depressive symptoms’ as having two or more symptoms, according to the MINI.

All values used to parameterize the values in COMMA were derived from Lifelines data. Variables that had been shown to be related to developing depressive symptoms, such as the amount of screen time per day, were not available in Lifelines’ COVID-19 questionnaires [39]. This means that these factors could not be included in COMMA without imputation or synthetic assumptions, potentially compromising COMMA’s validity.

For factors that were available in Lifelines, we used logistic regression to test whether the selected baseline characteristics and actions were related to the likelihood of developing depressive symptoms. Features and actions that were not significantly or substantively related to the likelihood of developing depressive symptoms were ultimately excluded from COMMA. This was the case with the features religion and migration background, and the actions willingness to comply with lockdown measures. Given that COMMA is already computationally intensive with a relatively long run-time, adding additional variables that did not meaningfully contribute to predicting depressive symptoms would have increased model complexity without improving explanatory power or model performance.

### The computational model in a nutshell

In COMMA, each lockdown type, either partial or full, agents had a series of probabilities of performing a series of actions, which in turn affected the probability of depressive symptoms. The model initially synthesized the population, then each agent was assigned a series of actions, based on their characteristics at baseline. The probabilities of these actions were determined at each step, simulating the daily actions of individuals. Based on the actions carried out by each agent, the model computed the risk of developing depressive symptoms. This computation took into account various factors, including the nature of the activities carried out in interaction with the individual characteristics of the agents. COVID-19 infection also played a role in the choice of actions, as it significantly restricted the number and types of actions for agents. Agents with COVID-19 infections could only stay at home until they recover. This restriction directly affected their daily activities and, consequently, their likelihood of developing depressive symptoms.

### The computational model in detail

#### Population synthesis

In COMMA, the first step generated agents, with features reflecting the Lifelines sample from baseline characteristics. The population composition also reflected Lifelines data that has been cross-tabulated. Taking gender and age as an example, the model accounted for the share of the sample who were men and women between the ages of 24 and 24, 35 and 44, 45 and 54, and 55 and 64. To handle missing data, we introduced an “unknown” category to nearly all variables, all of which were categorical. This approach was necessary because IPF relies on complete categorical distributions, and several variables had high levels of missingness that were unlikely to be missing at random (e.g. 45% of respondents did not provide income information).

To synthesize the population, we used Multidimensional Iterative Proportional Fitting (M-IPF) [40]. IPF is a procedure used to adjust the values in a data table to ensure that the sums of specific categories (like gender, age group, or income) match certain predetermined totals or “marginals” extracted in our case from Lifelines. When categories cannot represented in a 2 × 2 matrix (e.g., gender − 2 levels - by age group − 4 levels), it is best to use the Multidimensional variation of IPF which allows to deal with non-squared matrices. We implemented exactly this procedure with the package *mipfp* in R [41]. The output of this procedure was a table whereby agents are listed by row and their features by columns, along with their “weight,” i.e. how much each of characteristics should influence the overall distribution. A very low weight means that that particular combination was not common in the target population, while a higher weight indicated a more common or significant combination. The weight therefore reflected what was found to be very common or underrepresented in the population according to the marginal totals. We input this table into COMMA, and the software used the weights to generate a synthetic population that aligns with the tabular data from Lifelines. This was tested using chi^2^ tests.

#### Actions’ assignment to agents

COMMA’s second step determined the agents’ actions at each step, or day. To derive the probabilities of these actions, a series of logistic regressions were run. The probability of taking each action (coded as 1=action taken, 0=action not taken) was estimated using logistic regressions, with all baseline characteristics included as covariates. This is expressed as follows:

*logit(Pr(Y*_*i*_ *=1))=β*_*0*_ *+ β*_*1*_ *X 1*_*i*_ *+ β*_*2*_ *X *_*2i*_ *+ ⋯ +β*_*10*_ *X *_*10i*_

Where:

- *Y*_*i*_ is a binary outcome indicating whether individual *i* took a given action;
- *X*_*1i*_,*X*_*2i*_,*…*, *X*_*10i*_ are the baseline characteristics for individual *i*;
- *β*_*0*_ is the intercept;
- *β*_*1i*_, *β*_*2i*_,*…*,* β*_*10i*_ are the regression coefficients associated with each covariate.

In total, nine separate regressions (excluding contracting COVID-19, as COMMA handles this differently), one for each action, were run. Missing values of the outcome variables (actions) were imputed with multiple imputation. The resulting matrices of betas are reported in Appendix B (Tables B.1 and B.2). The goal of this matrix was to specify the likelihood of taking a particular action, given the agents’ set of features at baseline at a particular lockdown. We termed this type of matrix as the “lockdown probability matrix,” and we produced a matrix per lockdown type (partial or full). In every matrix, actions are listed by row, and all agents characteristics (one-hot encoded) by column. Each cell in this matrix has a beta reflecting a particular probability of choosing an action, depending on the agents features and the type of lockdown. Since the regressions were run using the Lifelines dataset, this step ensured that the probability of choosing an action was grounded in the data.

With this matrix of betas, the probability of performing an action *A* given the characteristics of the agents was then calculated. In order to do so, for every agent, we ran a series of matrix multiplications between the matrix resulting from the regressions run on the Lifelines data above, and a matrix reflecting the presence and absence of the agents characteristics (one-hot encoded). For instance, if an agent were male, and had an high education, then he would have a matrix containing 1 for the column “male,” 0 in “female,” and 1 in the column “education-high” but 0s in “education-low” and “education-medium.” The resulting matrix multiplication gave us for every action a beta for every characteristic present in that particular agent. The betas then were summed to have one value per action, per agent, per lockdown.

**Fig. 2:**
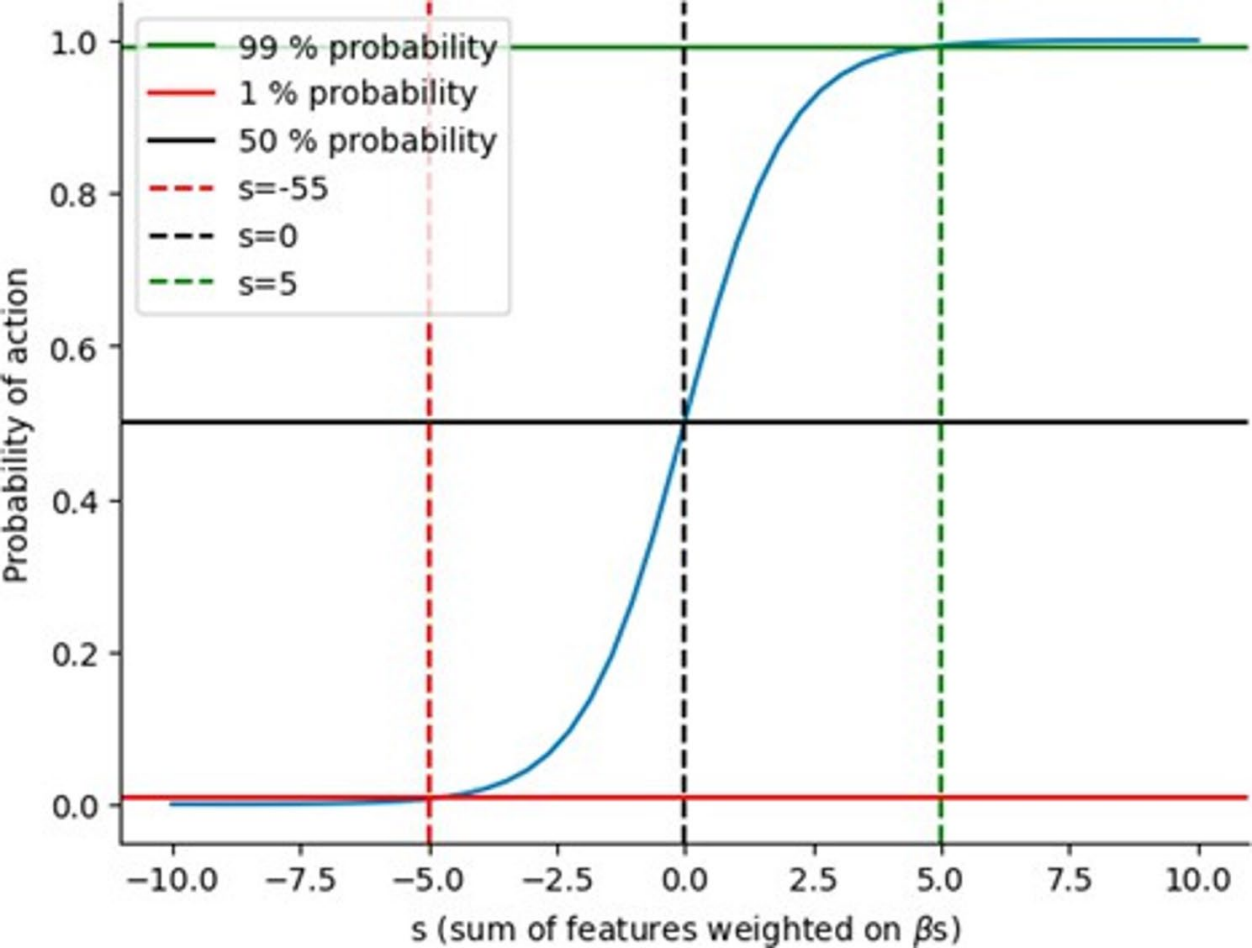
The probability of action *A* as a function of the sum in input to the sigmoid

Every beta per action per agent in the matrix was then transformed using a sigmoid function. This function ensured that the probability is a number between 0 and 1, giving a value close to 1 for large positive inputs, close to 0 for large negative inputs, and 0.5 for an input of 0. As shown in Fig. 2, the probability of action *A* varied as a function of the input to the sigmoid function. The input to the sigmoid function (Σ *β* × Feature) depended on the agent’s features, where, as described above, each feature was weighted by a beta. This is expressed as follows:

$$p\left(A\right)=\frac{1}{1+{e}^{-{{\Sigma}}_{i}{\beta}_{i}{x}_{i}}}$$

where:

- *p(A)* is the probability of performing action A;
- ${\beta}_{i}$ is the weight (or importance) of feature *i;*
- ${X}_{i}$ is the value of feature *i.*

Figure 2 illustrates this. For instance, if all betas were 0, *p(A)* was 0.5. When the sum of betas was around 5, the action was almost certain to happen (with approximately 99% probability), and when the sum was around − 5, the action was almost certain not to happen (with less than 1% probability). This approach assigned beta values to each feature, considering their contribution to making the action either almost impossible or certain, relative to a 0.5 chance baseline (a 50% probability). The output of this step was a probability of performing every action available (e.g. exercising, positive coping), per agent. This probability was determined by the characteristics of the agent (e.g. age, gender) and the type of lockdown in place. Once we calculated the probability of each action available during a specific lockdown, the next step was for the agent to decide whether to perform those actions.

We did this by sampling for each action a 1 or 0 based on the computed probability above, where a 1 (true) means the agent takes the action, and a 0 (false) means the agent does not take the action. This decision was made for each agent individually, and yielded a list of 1s and 0s indicating which action, for that specific day, a single agent was going to take.

#### The probability of depressive symptoms’ assignment to agents

Next, COMMA took the list of actions from the previous step, indicating the actions the agent is taking for that day (and lockdown), and estimates the effects of this action on his mental health, i.e., on the probability of developing depressive symptoms. To estimate this probability, similarly to the previous step, COMMA computed a probability that each action taken has a particular effect on increasing or decreasing the risk of developing depressive symptoms. The baseline effect on depressive symptoms when no action is taken or when action effects are cancelled out are drawn from a normal distribution with a mean of 0.002, with a standard deviation of 0.0005. Again, the probability was computed from betas obtained from a series of logistic regressions run on the Lifelines data. This time, feeling depressed was the dependent variable, a single action (e.g. working from home) was the key independent variable, and all agent features at baseline (wave 2 of Lifelines, prior to the COVID-19 pandemic) were included as covariates. This is expressed as follows:

*logit(Pr(Y*_*i*_ *=1))=β*_*0*_ *+ β*_*1*_ *X 1*_*i*_ *+ β*_*2*_ *X*_*2i*_ *+⋯+β*_*11*_ *X *_*11i*_

Where:

- *Y*_*i*_ is a binary outcome indicating whether individual *i* took a given action;
- *X*_*1i*_,*X*_*2i*_,*…*,*X*_*10i*_ are the baseline characteristics for individual *i*;
- *β*_*0*_ is the intercept;
- *β*_*1i*_ is the binary variable whether individual *i* took a given action;
- *β*_*2i*_,*…*,* β*_*11i*_ are the regression coefficients associated with each covariate.

In total, nine separate regressions, one for each action (excluding contracting COVID-19, as COMMA handles this differently), were run. Missing values of the outcome variable (depressive symptoms) were imputed with multiple imputation. The resulting betas indicate the magnitude and direction of the effects of taking a particular action on depressive symptoms. Separate regressions were run for full and partial lockdowns, resulting in a matrix of betas per lockdown with a beta per action, and per agent characteristic. The resulting matrices of betas are reported in Appendix B (Tables B.3 and B.4). The obtained betas were then used to compute the effects of performing a particular action on the risk of developing symptoms with matrix multiplications. Specifically, for every agent, we multiplied the betas of the matrix relative to the agents features which have been one-hot encoded (meaning that they will have only 1 or 0 for every characteristic listed in column) with the matrix of the betas relative to the actions effects on risk of developing depressive symptoms. This matrix multiplication was done only for the actions selected in the previous step. The resulting output was a matrix containing the actions chosen by row, and the betas relative to the effects of this actions on the agents by column. Every action had a beta for every agent characteristic (e.g. age, education, gender). These betas were summed and then transformed using a sigmoid function, as done in the previous step. This yielded a likelihood of having depressive symptoms at each step, or day, given the agents’ features and actions during lockdowns.

#### Assignment of positive COVID-19 cases and recovery

In COMMA, agents can also contract COVID-19. COVID-19 infection has been shown to affect brain function, and is associated with an increase in both depressive symptoms and major depressive disorder [42]. COVID-19 infection is assigned randomly, i.e. not based on agent features or actions. However, the proportion of agents each day who become infected is based on actual municipal COVID-19 case information [26]. The data were downloaded from the COVID-19 dashboard of RIVM, which lists the cumulative amount of positive individuals per day, from 2020 to 2022. When running the model, the user must select a particular time-period and a location from which gather these data. The length of the period that must be equal in number of days to the number of steps. Once the number of cumulative cases from the dashboard was obtained, we transformed this number to accommodate it to the size of the simulated population. We calculated the number of cases as:

$$cases=\frac{n}{N}\times{M}$$

where:

- *n* is the size of the simulated population;
- *N* is the size of the real population for the specific location;
- *M* is the number of new positives reported on RIVM.

When an agent is positive, the only action that agents can take is staying at home. This is hard coded in the model, meaning that when an agent is positive the models does not provide any choice of actions, but it forces probability of 1 to “stay at home”, and 0s to the rest. This rule in COMMA reflects real-world rules to stay at home and to not engage in social interactions during infection. In turn, staying at home increases the probability of developing depressive symptoms [43]. While this might be an overly simplistic rendering of reality, this results in agents with current COVID-19 infections to only experience an event that increases the likelihood of depressive symptoms.

We also implemented two types of recoveries: standard recovery from COVID-19 and recovery from long COVID-19. Having long COVID-19 has been found to increase the likelihood of experiencing depressive symptoms threefold [44]. While 80% of agents with COVID-19 experienced a standard recovery, 20% of positive agents have long COVID, based on estimates of long COVID-19 from the literature [45]. In the standard recovery, the agents stay positive for a minimum of 10 days (this number is fixed and hard-coded in the model), and the maximum depends on a gamma distribution that peaks at approximately 10 days and is determined by the parameters: a = 5 and scale = 3. In the long COVID scenario, the minimum recovery is 35 days, and the gamma distribution peaks at approximately 70 days, with parameters a = 9 and scale = 70/(a-1). The choice for the gamma distribution is based on the characteristic long-tailed distribution found in the literature [46].

### Simulation scenarios

In this study, COMMA was used to simulate the effect of three different lockdown scenarios on the probability of depressive symptoms for 1,000 individuals, in the period from 11 June 2020 to 11 June 2021. Each simulation was run 30 times, to account for stochastic variation. We report the average probabilities across runs, along with 95% confidence intervals. This sample size was chosen because it likely retained the key demographic structure of the Lifelines sample, while being able to be run on a laptop. The first simulation was designed to replicate what actually happened in terms of the lockdown policy. Here, there was a partial lockdown from 11 June 2020 to 3 November 2020, followed by a full lockdown from 4 November 2020 to 25 March 2021, and a partial lockdown from 26 March 2021 to 11 June 2021. Second, we ran a simulation in which there was only a partial lockdown for the entire year. Third, we simulated a year-long full lockdown. These results are reported in the main body of this paper.

Appendix C reports additional simulation results. We replicated this study’s main results, but varying: (1) the duration, instead running for 1 month and 6 months; (2) the timeframe, instead running from 1 February 2021 to 1 February 2022; (3) and the number of agents, using 10,000 agents. In most scenarios, we ran the simulation 30 times. In the case of the scenario using 10,000 agents, we only ran two simulations, due to computational intensity. We found strikingly similar results to those reported in the body of the paper, although longer lockdowns are associated with a slightly increased risk of experiencing depressive symptoms.

### Validation

To validate the model, we randomly reserved 30% of the Lifelines data as a “hold-out sample” [16]. We ran identical logistic regressions on both the 70% sample, and the 30% hold-out sample to estimate two sets of relationships: (1) baseline characteristics’ relationships to actions taken during lockdowns; and (2) actions taken during lockdowns’ relationships to depressive symptoms. The estimates from the 70% sample were used to populate the matrices, and are reported in Appendix B. After running COMMA, we assessed its validity by comparing the simulated outcomes to the regression-predicted outcomes from the 30% hold-out sample. We found that the results aligned closely with our main, reported results. We also compared the simulation results to the additional questionnaires, and also found that results aligned closely.

## Results

### Descriptive statistics

Descriptive characteristics at baseline can be found in Table A.2. Here, we also compared our sample to those who did not take part in any of the COVID-19 questionnaires. We found that our sample is older and more likely to be women than the full Lifelines sample. There was also much more missingness among those who did not participate in the COVID-19 questionnaires.

**Fig. 3:**
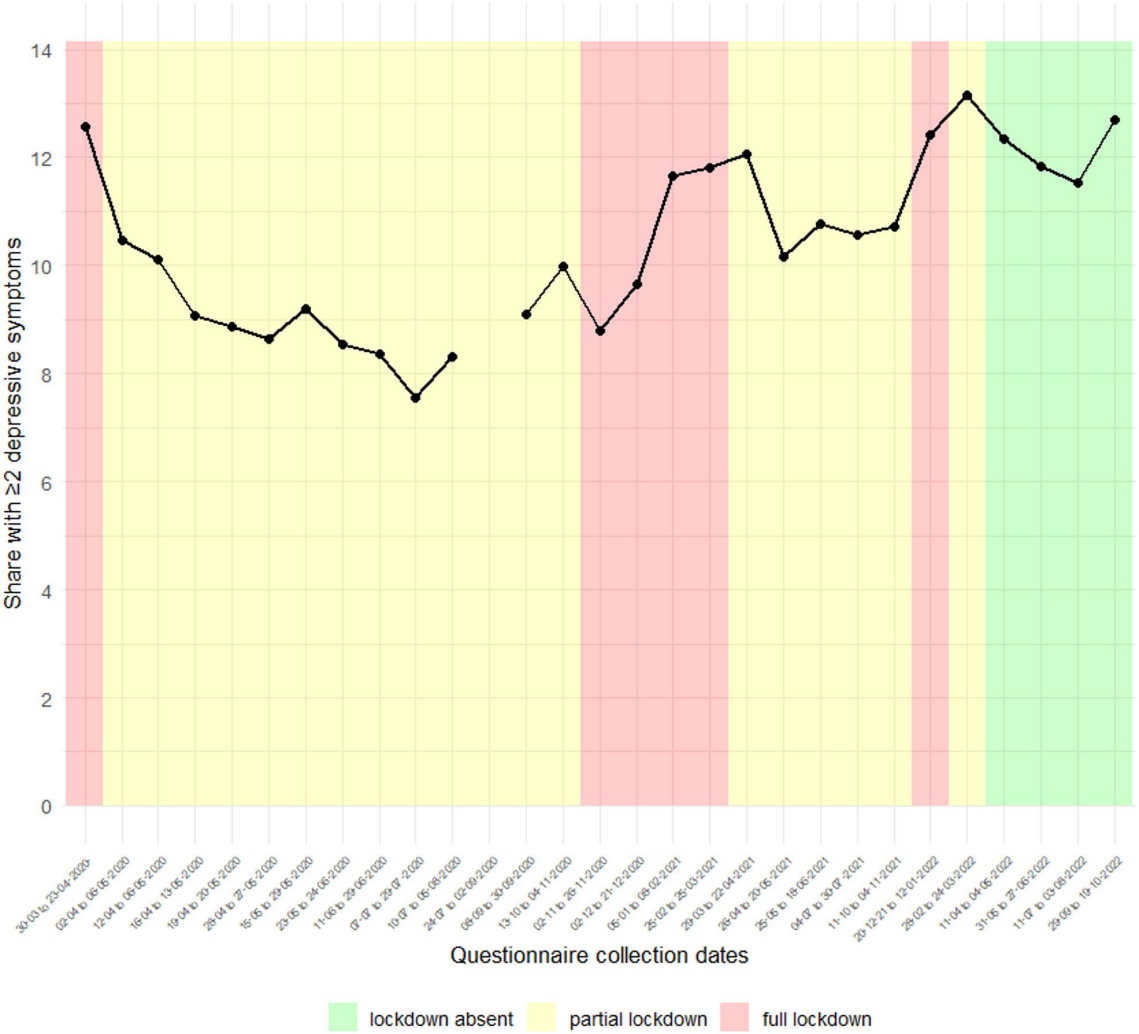
Percentage of participants with depressive symptoms by COVID-19 lockdown type

Figure 3 illustrates the relationship between lockdown severity and depressive symptoms. In most questionnaires, the share of participants saying they felt depressed (averaging 9.45% across questionnaires) was significantly different from the pre-COVID-19 period depressive symptom rate of 5.2%, based on paired-sample t-tests.

### Simulation results

**Fig. 4:**
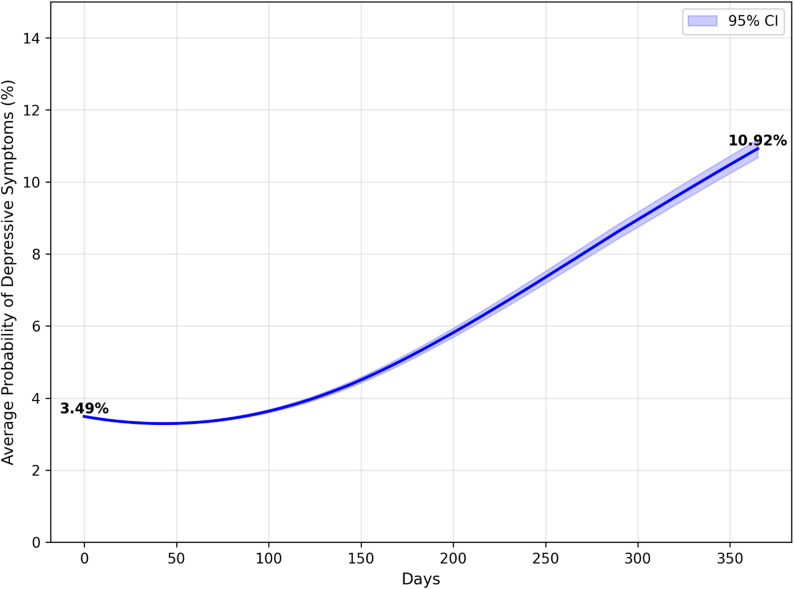
Probability of developing depressive symptoms in the actual lockdown scenario

**Fig. 5:**
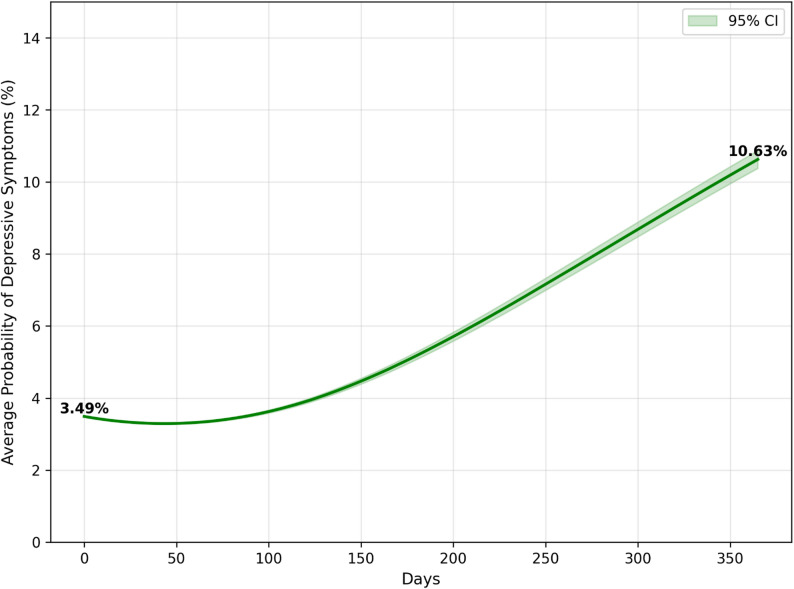
Probability of developing depressive symptoms in the partial lockdown scenario

**Fig. 6:**
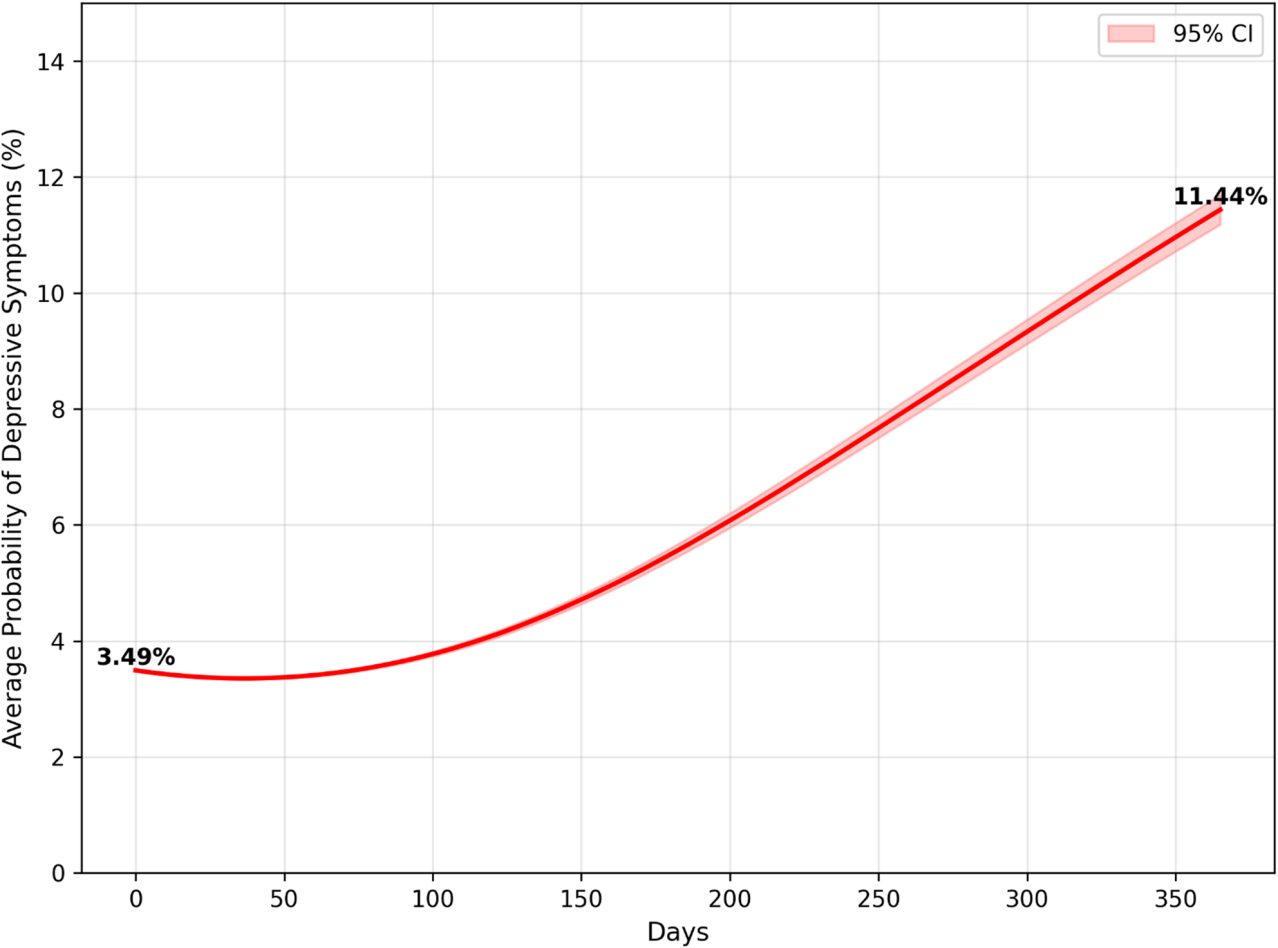
Probability of developing depressive symptoms in the full lockdown scenario

Figs 4, 5 and 6 present the results of one year in an actual, partial and full lockdown scenarios. After a year of actual lockdown scenarios, including a mix of partial and full lockdowns, the probability of having depressive symptoms was 10.92% (95% CI: 10.68%-11.18%), up from 3.49% (95% CI: 3.49–3.49) at baseline. This aligns closely with the observed depression rate of 10.77% in June 2021. In the hypothetical scenario of having only a partial lockdown in this period, the average probability of having depressive symptoms was 10.63% (95% CI: 10.38%-10.87%), or a 7.14% point increase from baseline. In the hypothetical scenario in which there was only a full lockdown in this period, the average probability of having depressive symptoms was 11.44% (95% CI: 11.18%-11.69%), or a 7.95% point increase from baseline. Although the actual lockdown scenario was not significantly different from hard and partial lockdowns, partial and hard lockdowns were significantly from one another.

In Appendix B, the parameters, or values, used in COMMA are reported. We also ran COMMA suppressing each baseline characteristic and action, to test their relative contribution to developing depression. Different actions during lockdown also were related the probability of developing depressive symptoms. We base these estimates on the individual action betas reported in tables B.3 and B.4. A positive beta is associated with an increased likelihood of experiencing depressive symptoms, and a negative beta with a decreased likelihood. In particular, exercising (β=-0.52 in a partial lockdown; β=-0.40 in a full lockdown), feeling socially connected (β=-0.51 in a partial lockdown; β=-0.30 in a full lockdown), and positive coping (β=-0.18 in a partial lockdown; β=-0.06 in a full lockdown) were associated with the biggest decreases in the risk of developing depressive symptoms. In contrast, feeling isolated (β = 0.68 in a partial lockdown; β = 1.18 in a full lockdown), being sedentary (β = 0.19 in a partial lockdown; β = 0.29 in a full lockdown), and drinking more than the weekly recommended alcoholic drinks (β = 0.15 in a partial lockdown; β = 0.38 in a full lockdown), or more drinks for women, and 14 or more drinks for men, per the National Institute on Alcohol Abuse and Alcoholism [47], were associated with the largest increases in the risks of developing depressive symptoms.

## Discussion

It is clear that COVID-19 lockdowns had a negative impact on depressive symptoms. However, it is not yet clear if different lockdown scenarios could have altered these outcomes. To that end, this study simulated the impact of COVID-19 lockdowns on depressive symptoms, allowing us to compare actual and hypothetical lockdown scenarios. COMMA was the first simulation model to account for different characteristics and actions that may make someone more or less susceptible to depressive symptoms as a result of COVID-19 lockdowns. This enabled us to more accurately estimate the impact of lockdowns may have had on depressive symptoms.

We found that all lockdown scenarios, partial or full and for any length of time, were associated with higher probabilities of depressive symptoms than prior to the COVID-19 pandemic. This aligns with research conducted in both the Netherlands and elsewhere [38, 48]. There was also a clear pattern in terms of lockdown types. Scenarios with full lockdowns only were associated with the largest share of the population who developed depressive symptoms, followed by scenarios with both full and partial lockdowns, and then by scenarios with partial lockdowns only. Lockdown type, rather than lockdown length, appeared to matter most in determining the prevalence of depressive symptoms. In periods when there was no reprieve from restrictions such as curfews and school closures, depressive symptoms were most prevalent [25]. These findings have clear public health implications: lockdowns substantially increased depressive symptoms among the Dutch population.

A number of mechanisms may explain these findings. First, in periods with full lockdowns, people were most likely to be spending most of their time at home, and were likely to have restrictions placed on travel and the number of social contacts they could have [25]. This was shown to increase loneliness and feelings of social isolation [49, 50]. Importantly, subjective assessments of isolation - e.g. feeling lonely, but having social contact - was more important than the actual number of social contacts, particularly during lockdowns [51]. This mechanism were accounted for in this study’s model, which includes actions during lockdowns such as feeling socially connected and feeling socially isolated, as well as the agent feature, living alone.

Second, particularly during full lockdowns, parents and other caregivers bore increased responsibility for their children’s education. This was especially the case during relatively long periods of online schooling [25]. Research has found that this increased stress and depression among parents, due to this greater workload [52]. We captured this by including having children as a baseline feature. This baseline feature was consistently associated with experiencing depressive symptoms during the COVID-19 pandemic, as well as with many actions that worsened mental health.

Still, we found that partial lockdowns also elevated the probabilities of depressive symptoms, albeit to a lesser extent than full lockdowns. Several factors present in both partial and full lockdowns likely explain this finding. In particular, the loss of health-promoting routines was a common feature across most partial and full lockdowns in the Netherlands. For instance, gyms were among the last businesses to reopen in the Netherlands at the end of the COVID-19 period, and were generally closed during partial and full lockdowns [25]. This resulted in a decrease in physical activity [53]. Physical activity is important for mental health, as it: “improves the functioning of the hypothalamus-pituitary-adrenal (HPA) axis, lowering cortisol secretion and restoring the balance of leptin and ghrelin” [54]. We accounted for health promoting behaviors in COMMA with actions such as getting more than 150 minutes per week of physical activity, and being sedentary for more than eight hours per day.

Additionally, in both partial and full lockdowns, people in the workforce would have likely have had their daily routines disrupted. For white collar workers, this would translate to working from home. For workers in essential sectors, such as front-line healthcare workers, the COVID-19 period was associated with much greater work pressures. For workers in customer-facing sectors, this sometimes resulted in a loss of income or loss of employment entirely [55]. People who were unemployed during the COVID-19 period felt increased rates of stress and depression [56, 57]. COMMA accounts for these different employment configurations by accounting for the action working from home, and for the baseline features of being in a critical sector, income, and education.

Somewhat curiously, we also found that the duration of lockdowns mattered less than their intensity. This aligns with a large body of literature that suggests mental health worsened in response to both shorter and longer lockdowns [58, 59]. Further, Probst et al. [60] found that, even after a shorter-term hard lockdown in Austria, rates of depression were still elevated. The authors argued that the short-term pressures associated with lockdowns likely did not dissipate immediately after the lockdowns ended [60].

This finding may also indicate that, over time, the restrictions placed on everyday life did not continue to increase the number of people experiencing depressive symptoms. Based on our findings, a large majority of the Dutch population were not at risk of developing depressive symptoms. Perhaps this is because most people are resilient to the negative mental health consequences of lockdowns. The literature around resilience indicates that most people are able to cope with extreme stressors, and are able to return to regular functioning after an initial shock [61]. There is evidence that this was also the case during the COVID-19 pandemic [14, 62]. COMMA captured this by including a measure of resilience as an action.

We also found that individuals with varying characteristics may be more or less at risk of experiencing depressive symptoms. Characteristics such as preexisting depression diagnosis and having housing and/or financial difficulties were associated with an increased risk of depressive symptoms. This is in line with existing research on risk factors during the COVID-19 period, and likely indicates an increased level of stress and susceptibility to depressive symptoms [63, 64]. Interestingly, being a younger adult was associated with an increased risk of depressive symptoms, while this has been found in a number of other studies [65, 66]. Perhaps this is because this study focused on adults from the age of 24, in order to focus on experiences of those in the labor force.

These results have implications for policy. First, the duration of lockdowns is less of a determinant of the effect on mental health than the type of lockdowns. Second, given that we found much lower rates of depressive symptoms in scenarios with partial lock-downs, or a mix of partial and full lockdowns, varying restrictions between partial and full lockdowns may also be an important way of preventing larger shares of the population becoming susceptible to depressive symptoms. Third, additional support for individuals with factors that predispose them to experiencing depressive symptoms - such as having a preexisting depression diagnosis, being in poor physical health, and living alone - may be important. Fourth, encouraging social connection and health-promoting behavior (e.g. exercise and physical activity), may also reduce the rate of individuals experiencing depressive symptoms. Groups with these baseline characteristics and behaviors may benefit from targeted interventions, if lockdowns in relation to COVID-19 or another infectious disease are being contemplated in the future [67].

### Limitations

This paper presents findings from an initial attempt at modelling depressive symptoms as a result of COVID-19 lockdowns. However, the model on which our findings are based, COMMA, had several limitations. First, this model does not including actions such as dying or becoming vaccinated. This was intentional, as we focused on understanding how surviving individuals’ mental health was affected by the pandemic and lockdowns. However, such information could be added to COMMA or similar models, to more accurately reflect individuals’ experiences of depressive symptoms during COVID-19 lockdowns.

Second, COMMA does not include periods without any lockdown restrictions during the COVID-19 pandemic. This is because of a lack of data availability. In the Netherlands, the only periods without lockdown restrictions during the COVID-19 period occurred nearly two years after the pandemic began. These periods saw elevated rates of depressive symptoms (based on Fig. 3) and are in all likelihood not representative of a scenario in which there was no lockdown at all. Further, the Lifelines questionnaires from these periods without lockdown restrictions did not contain many key variables used in this study.

Third, COMMA does not an include an autoregressive component for agent behavior. Each action is modeled independently at each time step, conditional on the agent’s baseline features and the lockdown type, without explicit reference to the agent’s prior actions. While this choice reduces model complexity, it may underestimate behavioral inertia or habit formation. For instance, someone who stays at home for several days may be more likely to do so on subsequent days. Incorporating time dependency into agent actions could more accurately reflect real-world behavior and dynamics.

Fourth, we encoded causal logic into COMMA. Based on the literature, we assumed that certain characteristics and actions cause an increased risk of depressive symptoms. However, we instead may have identified associations. For instance, being sedentary for more than eight hours per day may not cause depressive symptoms, and instead itself may be a cause of experiencing depressive symptoms. Caution is warranted in interpreting this study’s results.

### Conclusions

In this study, a microsimulation model, COMMA, was developed to explore different lockdown scenarios’ effects on depressive symptoms in the northern Netherlands. This is one of the first attempts to simulate the impacts of lockdowns on mental health. We found clear evidence that full lockdowns increased the prevalence of depressive symptoms, relative to partial lockdowns. Despite the modest increase in the prevalence of depressive symptoms across all scenarios, it appears that most individuals, regardless of the lockdown scenario, were unlikely to ever experience depressive symptoms. Rather, only particular population sub-groups with a particular set of features and who took certain actions during lockdowns were at risk of developing depressive symptoms. This indicates that most individuals in the population are resilient to the effects of lockdowns on depressive symptoms. These findings highlight that lockdowns are not without consequences for depressive symptoms.

## Supplementary Information

Below is the link to the electronic supplementary material.

Supplementary Material 1.

## Data Availability

The software developed as part of this study is available here: https://github.com/covid19ABM/comma. The data used to create the model’s synthetic population and the model parameters are available via the Lifelines Biobank, and cannot be shared openly due to patient privacy concerns.
