# Supplementary material for: Simulating COVID-19 lockdowns’ impact on depressive symptoms in the northern Netherlands
Source: BMC Public Health. 2026 Mar 21;26:1408. doi: 10.1186/s12889-026-27001-3 (PMC13126702; doi:10.1186/s12889-026-27001-3)
Supplement: Supplementary file 1 — Supplementary Material 1. [file 12889_2026_27001_MOESM1_ESM.pdf]

Appendices Table of Contents

Appendix A: Additional information on Lifelines……………………………………...p. 2
Appendix B: Matrices ……………………………………………………………..p. 9
Appendix C: Additional simulation results……………………………………………. p. 18

A. Additional information on Lifelines data

Table A.1: Lifelines Variables used in COMMA

Baseline characteristics used for agent features	Original question	Variable code
Depressed?	Depression / could you indicate which of the following disorders you have (had)?	depression_presence_adu_q_1
Gender	What is your gender?	GENDER
Age	What is your age?	AGE
Highest level of education	What is the highest level of education you have finished?	degree_highest_adu_q_1
Employment status	Which (employment) situation is most applicable?	employment_situation_adu_q_1
Partnership status	Do you have a partner now?	partner_presence_adu_q_1
Presence of children?	Who are living in your house? (more than half the time)	inhouse_children_adu_q_1<br>inhouse_stepchildren_adu_q_1_v1/2
Housing/financial trouble?	In the past year, to what extent did you experience difficulties and stress related to this aspect of your life?<br>home and living (e.g. accommodation too small, could not find a home, noise) finances (e.g. major debts, insufficient income)	ldi_housing_adu_q_01<br>ldi_finance_adu_q_09<br>rand_generalhealth_adu_q_01
Self-rated health	How would you rate your health, generally speaking?	

Essential job, based on the definition set by the Dutch government during the pandemic	job classification (code) according to isco 2008 for the profession named in profession_current_adu_q_1	work3a_iscocode
BMI category	Weight in KG and height in cm	bodylength_cm_all_m_1<br>bodyweight_kg_all_m_1
Live alone?	I live alone	inhouse_alone_adu_q_1
Monthly income	What is the net income per month?	income_net_adu_q_1_v3

**Actions during COVID-19 period**	**Original question**	**Variable code**
Feeling socially connected	I feel connected to my neighbors, family and/or friends (in the last 7/14 days)	connection_adu_q_1_b
Feeling isolated	How socially isolated have you felt in the last 7/14 days?	isolation_adu_q_1/2
Getting $\geq$ 150 minutes of physical activity per week	In the last 7/14 days, how many minutes of (moderately) intense activity did you do (e.g. walking, biking or running)?	activity_adu_q_1_b
Feeling resilient	I expect that I will learn something positive for my own life from the corona pandemic	positive_adu_q_1
Getting the help needed from family and friends	I get the help and support I need from my neighbors, family and/or friends (in the last 7/14 days)	support_adu_q_1
Being sedentary for $\geq$ 8 hours per day	In the past 7/14 days, how much time did you spend sitting, on average, per working day (Monday to Friday)?	sedentary_adu_q_1_c

Drinking more than the recommended number of alcoholic drinks per week (≥8 drinks for women, and ≥14 units for men)	How many glasses of alcohol did you drink (in total) in the past 7/14 days?	alcohol_adu_q_2-4
Maintaining physical distance from others	I do not feel obliged to comply with the government's corona measures (in the last 7/14 days)	society_adu_q_1_a
Working from home	On average, how many hours per week do you work from home?	workhome_adu_q_2

**Measure of depressive symptoms during COVID-19 period**	**Original question**	**Variable code**
≥ 2 depressive symptoms	In the last 7/14 days have you felt low or depressed for much of the day, every day? In the last 7/14 days have you had the feeling that you've lost interest in or the will to do things you are normally interested in? Did your appetite change noticeably, or did your weight increase or decrease without this being intended? (In the last 7/14 days) Have you had problems sleeping almost every night (difficulty falling asleep, waking up in the night or too early in the morning, or actually sleeping too much)? (In the last 7/14 days)	minia1_adu_q_1/2<br>minia2_adu_q_1/2<br>minia3a_adu_q_1/2<br>minia3b_adu_q_1/2

	Did you speak or move more slowly than normal? Or did you feel restless, jittery and could barely sit still? Nearly every day? (In the last 7/14 days)	minia3c_adu_q_1/2
	Did you feel worthless or guilty almost every day? (In the last 7/14 days)	minia3e_adu_q_1/2 minia3f_adu_q_1/2
	Was it difficult to concentrate or make decisions almost every day? (In the last 7/14 days)	minia3g_adu_q_1/2
	Have you considered hurting yourself, wished you were dead, or had suicidal thoughts? (In the last 7/14 days)	

Table A.2: Descriptive statistics of baseline sample, comparing those who participated in the COVID-19 questionnaires with those who did not

	COVID-19 participants (n=52378)		Non-COVID-19 participants (n=33334)	
**Variable**	**n**	**%**	**n**	**%**
**Gender**				
Female	32443	61.94	18512	55.53
Male	19935	38.06	14822	44.47
**Age**				
24-34	5976	11.41	5919	17.76
35-44	11459	21.88	9409	28.23
45-54	22497	42.95	13232	39.70
55-64	12446	23.76	4774	14.32
**Highest level of education**				
Low	9544	18.22	7002	21.01
Middle	16680	31.85	10160	30.48
High	16229	30.98	7945	23.83
Unknown	9925	18.95	8227	24.68
**Employment status**				
Employed	46009	87.84	27320	81.96
Unemployed	1226	2.34	1493	4.48
Unknown	5143	9.82	4521	13.56
**Partnership status**				
Single	5223	9.97	3176	9.53
Partner	42078	80.34	24839	74.52
Unknown	5077	9.69	5319	15.96
**≥2 depressive symptoms in the past 2 years?**				
No	41410	79.06	24191	72.57
Yes	2681	5.12	1852	5.56
Unknown	8287	15.82	7291	21.87
**Children's presence in household**				
No children	21846	41.71	10982	32.95
Kids (step or bio) in house	25906	49.46	17324	51.97
Unknown	4626	8.83	5028	16.74
**Housing/financial trouble?**	7			
No	38761	74.00	21541	64.62

Table A.2 – continued from previous page

Variable	n	%	n	%
Yes	10030	19.16	6212	18.64
Unknown	3587	6.85	5581	16.74
**Self-rated health**				
Good/very good health	17365	33.15	8940	26.82
Average health	27563	52.62	16313	48.94
Poor/very poor health	3979	7.60	2602	7.81
Unknown	3471	6.63	5581	16.44
**Employed in an essential sector?**				
No	20920	39.94	11867	35.60
Yes	18449	35.22	11064	33.19
Unknown	13009	24.89	10403	31.21
**BMI category**				
Underweight	434	0.83	240	0.72
Normal weight	23998	45.82	15063	45.19
Overweight	20032	38.25	12794	38.38
Obese	7853	14.99	5197	15.59
Unknown	61	0.12	40	0.12
**Living situation**				
Lives with others	42858	81.82	25550	76.65
Lives alone	4894	9.34	2756	8.27
Unknown	4626	8.83	5028	15.08
**Household monthly income**				
Below	12076	23.06	4172	12.52
Above	16294	31.11	4863	14.59
Unknown	24008	45.84	24299	72.90

B. Matrices

B.1 Matrix specifying the likelihood of taking a particular action, given the agents' set of features at baseline during a partial lockdown (results of logistic regressions; each column includes results of a separate logistic regression with that action as the outcome)

	**Actions**								
**Features**	**work_from_home**	**stay_home**	**exercise**	**feel_socially_connected**	**seek_help_from_friends**	**heavy_drinking**	**positive_coping**	**feel_isolated**	**be_sedentary**
baseline	-0.16	2.86	0.22	1.57	0.1	-2.3	-0.54	0.12	-0.9
gender_M	-0.02	-0.51	-0.01	-0.39	-0.17	-0.43	-0.19	-0.5	0.03
gender_F									
age_group__1	0.11	-0.25	-0.44	-0.5	0.31	-0.21	0.11	0.3	0.1
age_group__2	0.14	-0.32	-0.15	-0.18	0.03	-0.22	0.09	0.01	-0.05
age_group__3									
age_group__4	0.01	0.09	0.1	0.57	0.17	0.19	-0.03	0.11	-0.33
education_Low	-0.72	-0.16	-0.19	0.02	0.02	0.06	-0.23	-0.11	-0.16
education_Medium									
education_High	1.35	0.37	0.13	0.19	0.07	0.12	0.29	-0.02	0.49
education_unknown	0.62	0	-0.01	0.4	0.09	-0.03	0.03	-0.02	0.27
Unemployed_yes	-0.58	-0.12	0.13	-0.25	-0.29	0.7	-1	0.9	0.23
Unemployed_no									
Have_partner_no									
Have_partner_yes	-0.2	0.44	0.13	0.13	-0.12	0.1	0.3	-0.15	-0.26
Have_partner_unknown	-0.1	-0.49	-0.06	-0.19	-0.05	0.14	0.7	0	-0.43
Depressed_yes	0.04	-0.19	-0.11	-0.41	-0.28	1	-0.9	0.68	0.22
Depressed_no									
Depressed_unknown	0	-0.07	-0.01	-0.32	0.1	0.1	0.02	0.02	0
Children_presence_yes	0.01	0.19	-0.01	0.12	0.06	-0.21	0.03	-0.07	-0.13
Children_presence_no									
Children_presence_unknown	-0.01	0	0.01	-0.03	0.05	0.00	-0.01	-0.04	0
housing_financial_difficulties_yes	0.1	-0.1	-0.12	-0.27	-0.12	1.28	0.13	0.13	0.12

housing_financial_difficulties_no									
housing_financial_difficulties_unknown	0.13	-0.87	-0.04	0.85	-0.1	0.35	-0.12	0.21	0.21
selfrated_health_good	0.04	0	0.24	0.15	-0.05	0.07	0.12	-0.22	-0.07
selfrated_health_average									
selfrated_health_poor	0.14	-0.14	-0.24	-0.32	-0.5	-0.05	-0.11	0.28	0.27
selfrated_health_unknown	0.08	0.095	0.06	-0.66	0.05	-0.11	0.1	-0.02	-0.1
critical_job_yes	-0.84	0.04	-0.07	0.02	0.04	-0.21	-0.06	0.04	-0.37
critical_job_no									
critical_job_unknown	0	-0.21	0.08	0	0	-0.26	0	0.29	0.14
bmi_underweight	-0.11	-0.36	0.25	-0.03	0.2	-0.23	-0.14	-0.11	0.14
bmi_normalweight									
bmi_overweight	0	0.05	-0.25	-0.02	0.05	-0.02	-0.01	0.07	0.5
bmi_obese	-0.03	0.01	-0.54	-0.2	0	-0.29	-0.08	0.15	0.22
bmi_unknown	0.12	0	-0.02	0	0.04	0	0.05	-0.07	0.06
livesalone_no									
livesalone_yes	-0.05	-0.09	0.02	-0.04	0.12	0.37	0.11	-0.03	0.08
livesalone_unknown	0	-0.20	-0.04	0.05	0.20	-0.20	0.04	-0.03	0.03
income_median_below									
income_median_above	0.4	0.3	0.12	0.01	0.06	0.18	0.16	-0.02	0.37
income_median_unknown	0.03	-0.06	0.04	-0.2	-0.05	-0.42	-0.11	0.02	0.07

B.2 Matrix specifying the likelihood of taking a particular action, given the agents' set of features at baseline during a full lockdown (results of logistic regressions; each column includes results of a separate logistic regression with that action as the outcome)

	Actions								
**Features**	**work_from_home**	**stay_home**	**exercise**	**feel_socially_connected**	**seek_help_from_friends**	**heavy_drinking**	**positive_coping**	**feel_isolated**	**be_sedentary**
baseline	-0.08	2.44	0.01	0.88	-0.41	-1.47	-0.98	0.47	0.01
gender_M	-0.03	-0.47	0.01	-0.35	-0.16	-0.01	-0.1	-0.45	0.05
gender_F									
age_group__1	0.19	-0.66	-0.54	-0.34	0.54	-0.46	0.12	0.35	0.21
age_group__2	0.18	0.03	-0.2	-0.2	0.18	-0.23	0.08	0.03	0.18
age_group__3									
age_group__4	-0.41	0.05	0.27	0.52	0.08	0.35	-0.16	0.1	-0.1
education_Low	-0.73	-0.16	-0.18	-0.09	0.07	-0.04	-0.21	-0.12	-0.3
education_Medium									
education_High	1.21	0.02	0.25	0.42	0.07	0.32	0.44	-0.07	0.57
education_unknown	0.63	0.12	0.07	0.15	0.01	0.11	0.24	-0.09	0.23
Unemployed_yes	-0.55	-0.5	-0.5	-1.19	-0.3	0.58	-0.4	0.6	0.11
Unemployed_no									
Have_partner_no									
Have_partner_yes	-0.19	0.05	0.02	-0.23	-0.06	0.15	0.15	0.01	-0.24
Have_partner_unknown	-0.3	-0.16	-0.48	-0.54	-0.06	0.51	0.24	-0.09	-0.17
Depressed_yes	0.13	-0.13	-0.24	-1.23	-1.07	1	-1.4	1.42	0.15
Depressed_no									
Depressed_unknown	0.04	-0.06	0.01	0.07	-0.02	-0.12	0.06	0.12	-0.01
Children_presence_yes	0.11	-0.14	0.05	0.09	0.13	-0.15	0.05	-0.06	-0.12
Children_presence_no									
Children_presence_unknown	0	0	0	0	0	0	0	0	0
housing_financial_difficulties_yes	0.02	-0.2	-0.13	-0.06	-0.15	0.1	-0.41	0.17	0.13

housing_financial_difficulties_no									
housing_financial_difficulties_unknown	0.21	-0.28	0.2	0.2	-0.1	0.2	-0.2	0	-0.28
selfrated_health_good	0.08	-0.12	0.23	0.17	0.06	0.14	0.32	-0.24	-0.01
selfrated_health_average									
selfrated_health_poor	0.04	0.03	-0.17	-0.34	0.05	-0.14	-0.09	0.27	0.16
selfrated_health_unknown	-0.14	-0.03	-0.16	-0.05	0.13	-0.05	-0.17	0.07	0.03
critical_job_yes	-1.3	0.19	0.06	0.05	0.03	-0.05	-0.09	0.04	-0.34
critical_job_no									
critical_job_unknown	0	0	0	0	0	0	0	0	0
bmi_underweight	0.26	-0.08	0.82	0.33	-0.15	-0.14	0.21	-0.15	-0.11
bmi_normalweight									
bmi_overweight	0.02	-0.15	-0.23	-0.07	0.08	0.2	-0.09	0.03	0.27
bmi_obese	0.1	-0.28	-0.57	-0.08	0.08	0.39	-0.17	0.1	0.52
bmi_unknown	-0.04	-0.05	-0.07	0.03	0.06	-0.07	0.5	-0.69	0.08
livesalone_no									
livesalone_yes	-0.14	0.16	-0.06	-1.28	0.24	0.09	0	0.05	-0.05
livesalone_unknown	0	0	0	0	0	0	0	0	0
income_median_below									
income_median_above	0.39	0.02	0.15	0.17	0.01	0.2	0.13	-0.09	0.29
income_median_unknown	0.07	0.17	0.07	-0.12	-0.11	-0.16	-0.19	-0.02	-0.21

B.3 Matrix specifying the likelihood of experiencing depressive symptoms, given the agents' actions set of features at baseline during a partial lockdown (results of logistic regressions; each column includes results of a separate logistic regression with that action as an independent variable)

	**Actions**								
**Features**	**work_from_home**	**stay_home**	**exercise**	**feel_socially_connected**	**seek_help_from_friends**	**heavy_drinking**	**positive_coping**	**feel_isolated**	**be_sedentary**
Action's beta	-0.06	0.07	-0.52	-0.51	-0.24	0.15	-0.18	0.68	0.19
gender_M	-0.45	-0.45	-0.45	-0.47	-0.46	-0.4	-0.36	-0.34	-0.18
gender_F									
age_group__1	0.21	0.21	0.18	0.17	0.21	0.32	0.2	0.25	0.2
age_group__2	0.21	0.21	0.2	0.21	0.22	0.21	0.22	0.2	0.14
age_group__3									
age_group__4	-0.46	-0.46	-0.45	-0.42	-0.44	-0.46	-0.32	-0.46	-0.39
education_Low	0	0	-0.02	-0.01	0	0.07	0.03	0.07	-0.03
education_Medium									
education_High	0.02	0.01	0.01	0.02	0.02	0.07	-0.05	0.08	-0.02
education_unknown	0.04	0.03	0.03	0.04	0	0.02	-0.12	0.17	0.08
Unemployed_yes	0.35	0.35	0.35	0.38	0.4	0.4	0.35	0.36	0.39
Unemployed_no									
Have_partner_no									
Have_partner_yes	-0.24	-0.33	-0.42	-0.34	-0.46	-0.21	-0.11	-0.17	-0.26
Have_partner_unknown	0.04	0.04	0.03	0.03	0.04	-0.01	0.1	-0.11	-0.17
Depressed_yes	0.71	0.71	0.71	0.71	0.72	0.65	0.7	0.62	0.7
Depressed_no									
Depressed_unknown	0.02	0.02	0.02	0.03	0.04	0.13	0.19	0.13	0.1
Children_presence_yes	0.08	0.08	0.08	0.09	0.08	-0.01	0.1	-0.01	0.07
Children_presence_no									
Children_presence_unknown	-0.03	-0.03	-0.03	-0.02	-0.03	-0.01	-0.03	0	-0.01
housing_financial_difficulties_yes	0.65	0.65	0.65	0.64	0.64	0.62	0.55	0.59	0.6
housing_financial_difficult									

ies_no									
housing_financial_difficulties_unknown	0.24	0.24	0.24	0.22	0.22	0.12	0.4	0.1	0.46
selfrated_health_good	-0.47	-0.47	-0.45	-0.46	-0.47	-0.52	-0.48	-0.48	-0.58
selfrated_health_average									
selfrated_health_poor	0.99	0.99	0.98	0.98	0.99	0.89	0.78	0.89	0.92
selfrated_health_unknown	0.62	0.62	0.62	0.65	0.63	0.45	0.16	0.46	0.03
critical_job_yes	0	0.04	0	0.02	0.02	0.1	0.09	0.08	0.05
critical_job_no									
critical_job_unknown	0.01	0.01	0.01	0.01	0.01	0.01	0.01	0.01	0.01
bmi_underweight	0.01	0.01	0.03	0	0	0.21	0.23	0.22	0.3
bmi_normalweight									
bmi_overweight	0.12	0.12	0.11	0.12	0.13	0.1	0.13	0.1	-0.07
bmi_obese	0.71	0.71	0.22	0.26	0.26	0.25	0.2	0.2	0.11
bmi_unknown	0.07	0.07	0.06	0.06	0.07	-0.14	-0.05	-0.08	-0.06
livesalone_no									
livesalone_yes	0.23	0.23	0.23	0.22	0.22	0.17	0.25	0.06	0.33
livesalone_unknown	0.08	0.08	0.08	0.1	0.09	0.14	0.14	0.09	0.1
income_median_below									
income_median_above	-0.17	-0.17	-0.17	-0.16	-0.16	-0.17	-0.16	-0.16	-0.02
income_median_unknown	-0.19	-0.19	-0.19	-0.19	-0.22	0.2	-0.16	-0.18	-0.33

B.4 Matrix specifying the likelihood of experiencing depressive symptoms, given the agents' actions set of features at baseline during a full lockdown (results of logistic regressions; each column includes results of a separate logistic regression with that action as an independent variable)

	**Actions**								
**Features**	**work_from_home**	**stay_home**	**exercise**	**feel_socially_connected**	**seek_help_from_friends**	**heavy_drinking**	**positive_coping**	**feel_isolated**	**be_sedentary**
Action's beta	0.08	0.21	-0.4	-0.3	-0.04	0.38	-0.06	1.18	0.29
gender_M	-0.31	-0.18	-0.31	-0.34	-0.31	-0.14	-0.29	-0.3	-0.36
gender_F									
age_group__1	0.25	0.22	0.2	0.34	0.25	0.25	0.18	0.24	0.31
age_group__2	0.19	0.16	0.18	0.18	0.2	0.16	0.06	0.15	0.15
age_group__3									
age_group__4	-0.49	-0.39	-0.46	-0.46	-0.49	-0.41	-0.5	-0.5	-0.48
education_Low	0.08	0.03	0.06	0.1	0.08	-0.04	0.1	0.14	0.17
education_Medium									
education_High	-0.07	-0.02	-0.03	0.04	-0.05	-0.02	0.08	0.02	0.03
education_unknown	0	0.17	0.01	-0.06	0	0.21	-0.01	-0.07	-0.09
Unemployed_yes	0.5	0.51	0.4	0.43	0.52	0.5	0.52	0.58	0.47
Unemployed_no									
Have_partner_no									
Have_partner_yes	-0.01	-0.02	-0.02	0	-0.59	-0.2	-0.23	-0.06	-0.08
Have_partner_unknown	0.09	-0.12	0.06	-0.06	0.03	-0.03	-0.01	0.03	0.02
Depressed_yes	0.86	0.81	0.84	0.79	0.86	0.81	0.88	0.77	0.84
Depressed_no									
Depressed_unknown	0.2	0.06	0.2	0.16	0.21	0.01	0.19	0.15	0.2
Children_presence_yes	0.07	0.05	0.08	0.12	0.08	0.05	0.06	0.13	0.12
Children_presence_no									
Children_presence_unknown	0.03	0	0.03	0.01	0	0	0.01	0.01	0.01
housing_financial_difficulties_yes	0.5	0.6	0.49	0.44	0.49	0.6	0.63	0.41	0.45
housing_financial_difficulties_no									

housing_financial_difficulties_unknown	-0.11	0.37	0.12	0.12	-0.19	0.38	0	0.21	0.15
selfrated_health_good	-0.52	-0.6	-0.5	-0.52	-0.52	-0.59	-0.56	-0.48	-0.53
selfrated_health_average									
selfrated_health_poor	0.81	0.96	0.8	0.78	0.81	0.96	0.96	0.72	0.78
selfrated_health_unknown	0.52	0.21	0.49	0.34	0.6	0.2	0.53	0.31	0.31
critical_job_yes	0.15	0.14	0.14	0.09	0.14	0.14	0.24	0.08	0.13
critical_job_no									
critical_job_unknown	0.01	0.01	0.03	0	0	0.03	0.08	0	0.11
bmi_underweight	0.35	0.37	0.4	0.4	0.36	0.37	0.28	0.14	0.08
bmi_normalweight									
bmi_overweight	0.1	-0.05	0.08	0.11	0.1	-0.05	0.16	0.07	0.06
bmi_obese	0.15	0.11	0.08	0.08	0.15	0.12	0.25	0.01	-0.04
bmi_unknown	0.02	0	0	0.01	0	0.03	0	0	0.01
livesalone_no									
livesalone_yes	0.2	0.3	0.2	0.21	0.22	0.3	0.36	0.26	0.2
livesalone_unknown	0.02	0.02	0.02	-0.01	0.02	0.03	0.02	0.02	0
income_median_below									
income_median_above	-0.04	-0.02	-0.02	-0.07	-0.04	0	-0.06	0.09	0.05
income_median_unknown	-0.21	-0.26	-0.19	-0.1	-0.22	-0.26	-0.09	-0.08	-0.03

C. Additional simulation results

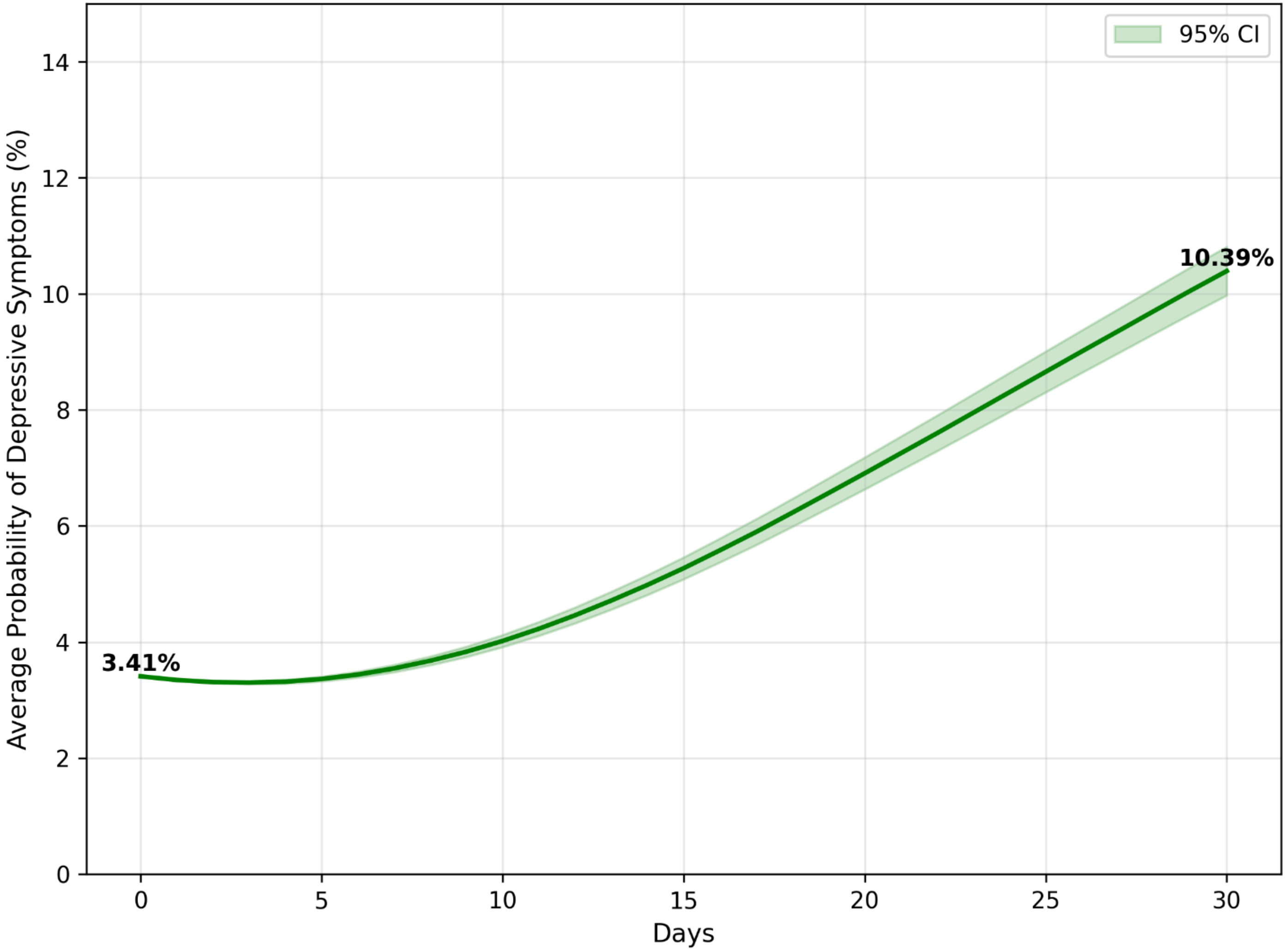

Figure A.1: Probability of developing depressive symptoms in the actual lockdown scenario, 1 month duration

(NB: this is the same as the partial lockdown scenario)

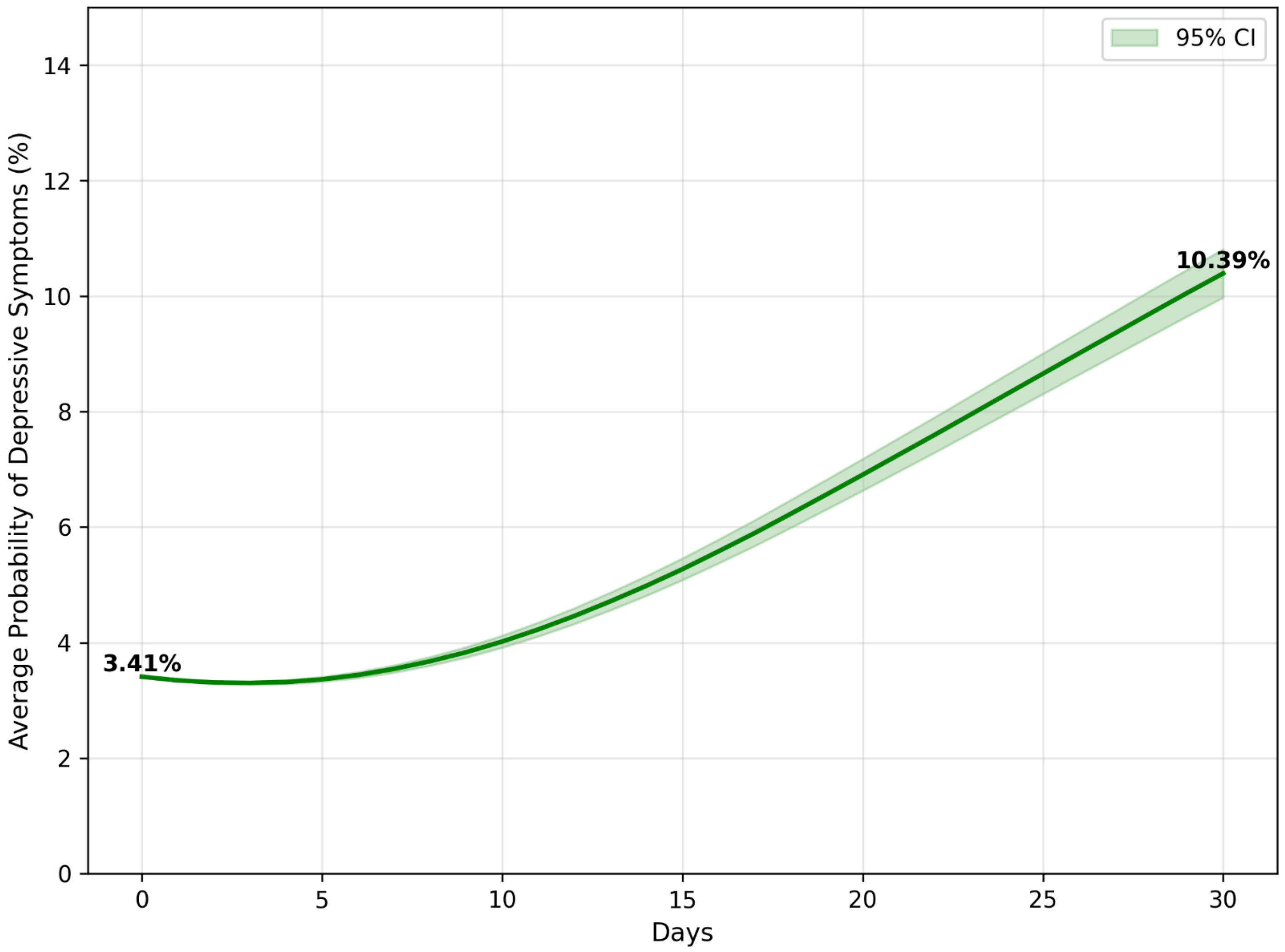

Figure A.2: Probability of developing depressive symptoms in the partial lockdown scenario, 1 month duration

(NB: this is the same as the actual lockdown scenario)

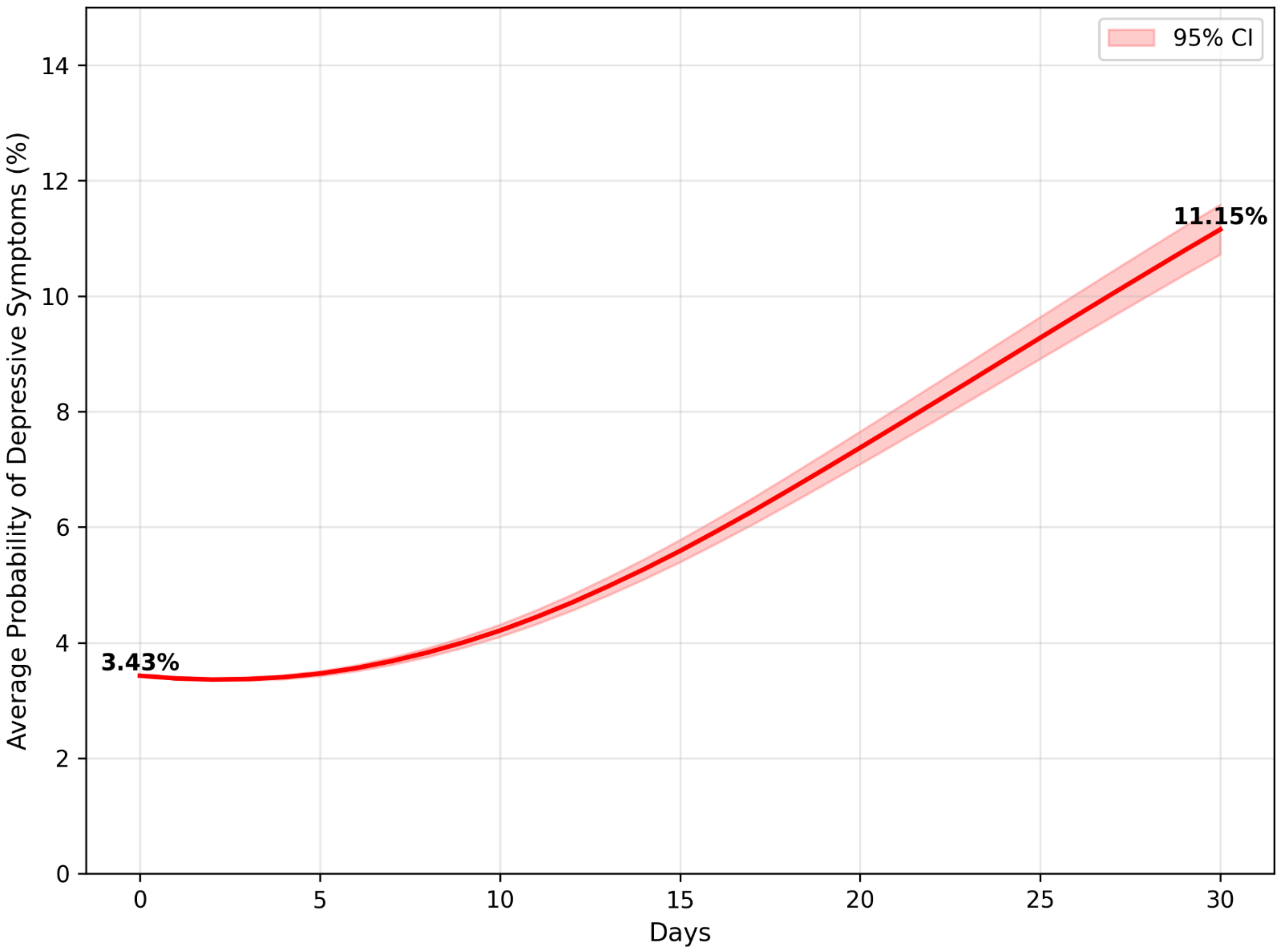

Figure A.3: Probability of developing depressive symptoms in the full lockdown scenario, 1 month duration

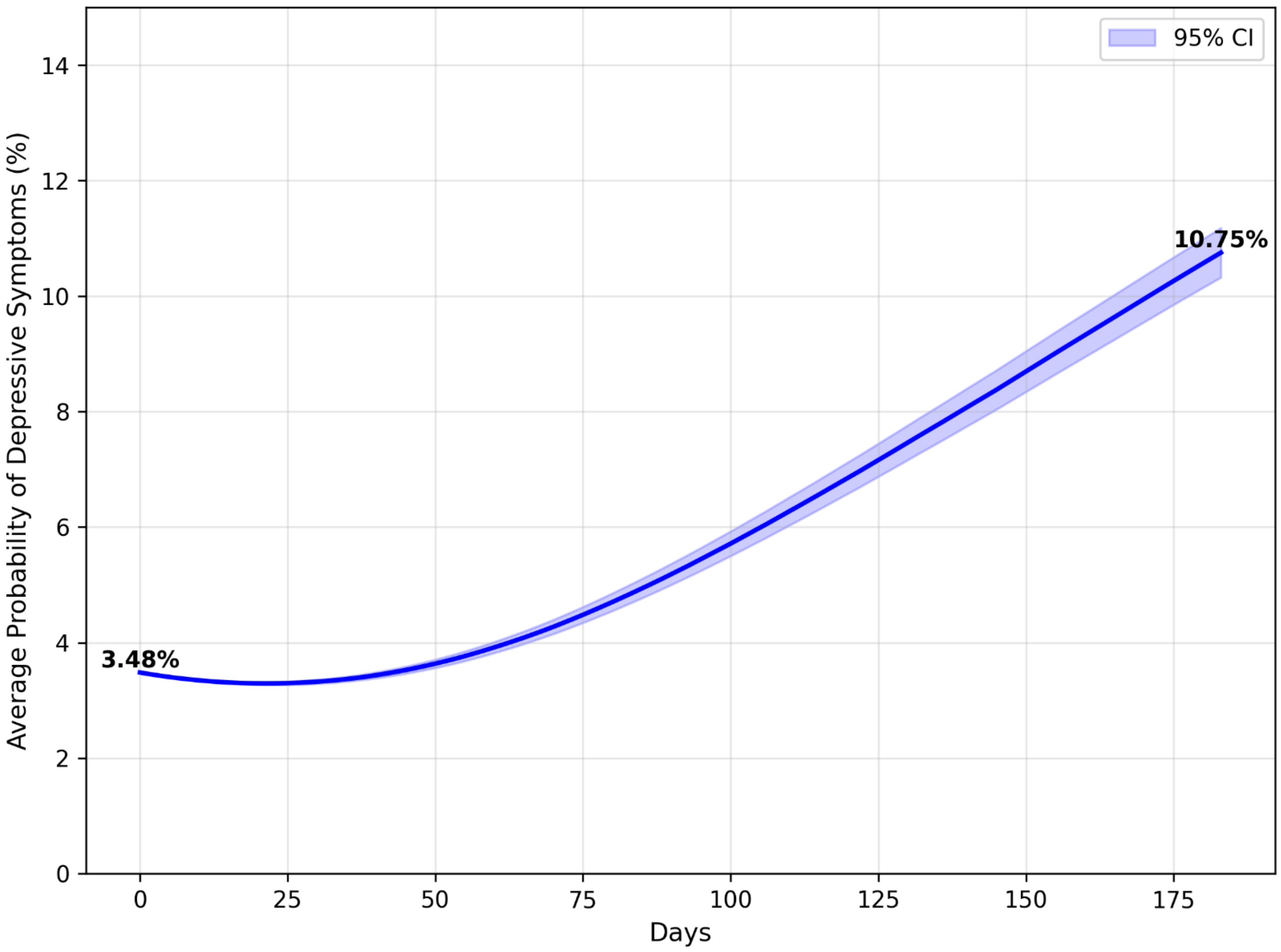

Figure A.4: Probability of developing depressive symptoms in the actual lockdown scenario, 6 month duration

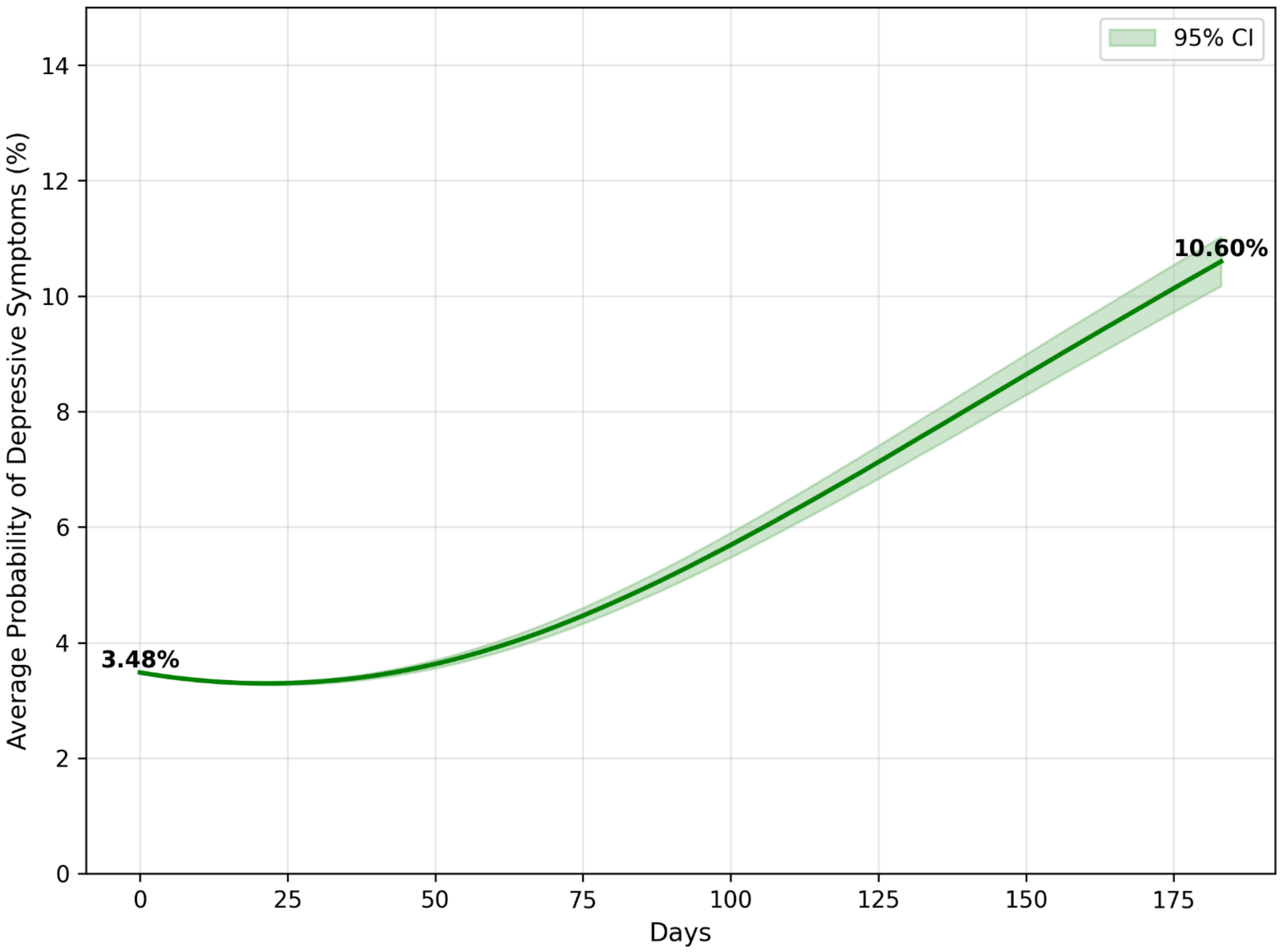

Figure A.5: Probability of developing depressive symptoms in the partial lockdown scenario, 6 month duration

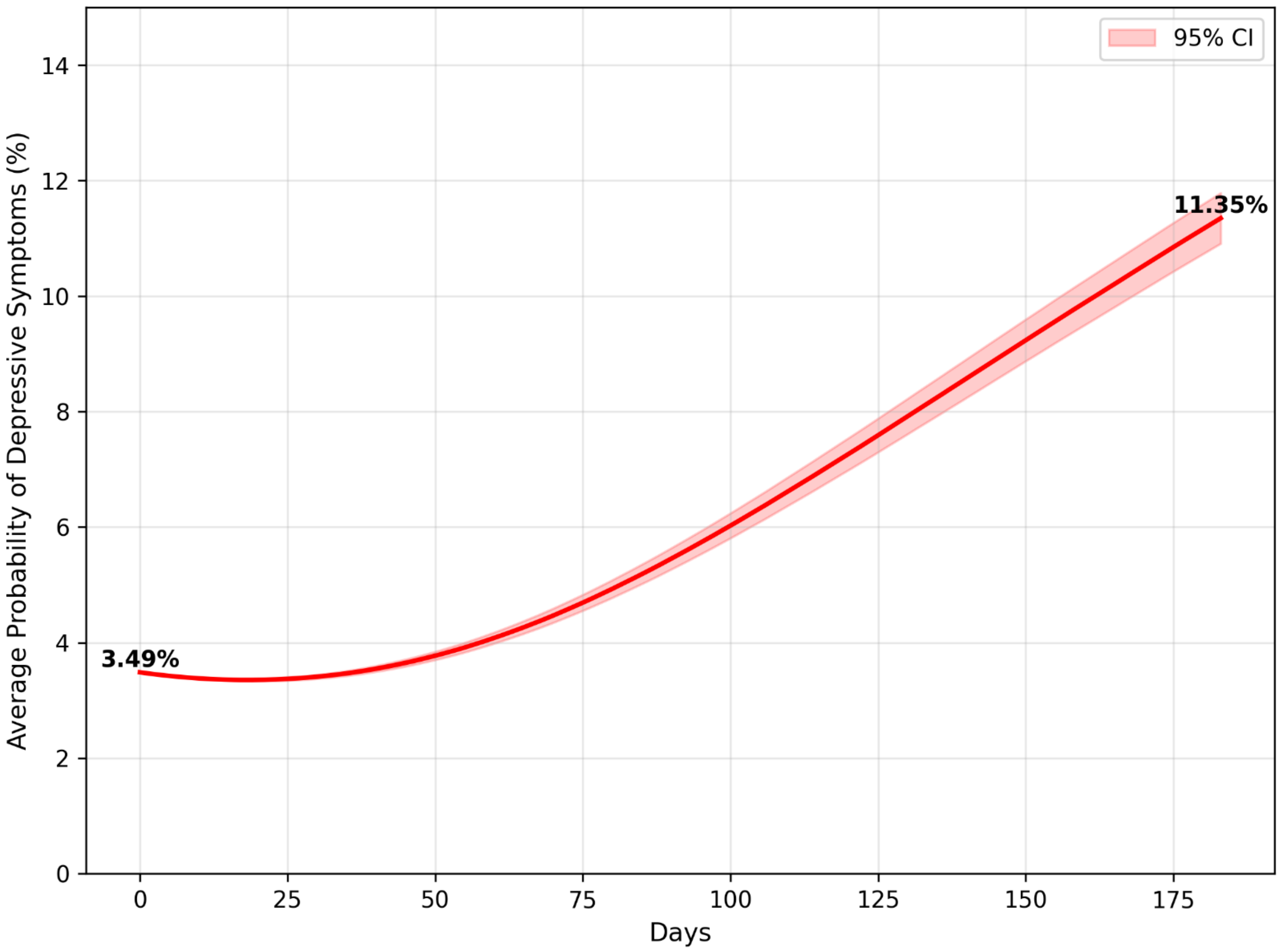

Figure A.6: Probability of developing depressive symptoms in the full lockdown scenario, 6 month duration

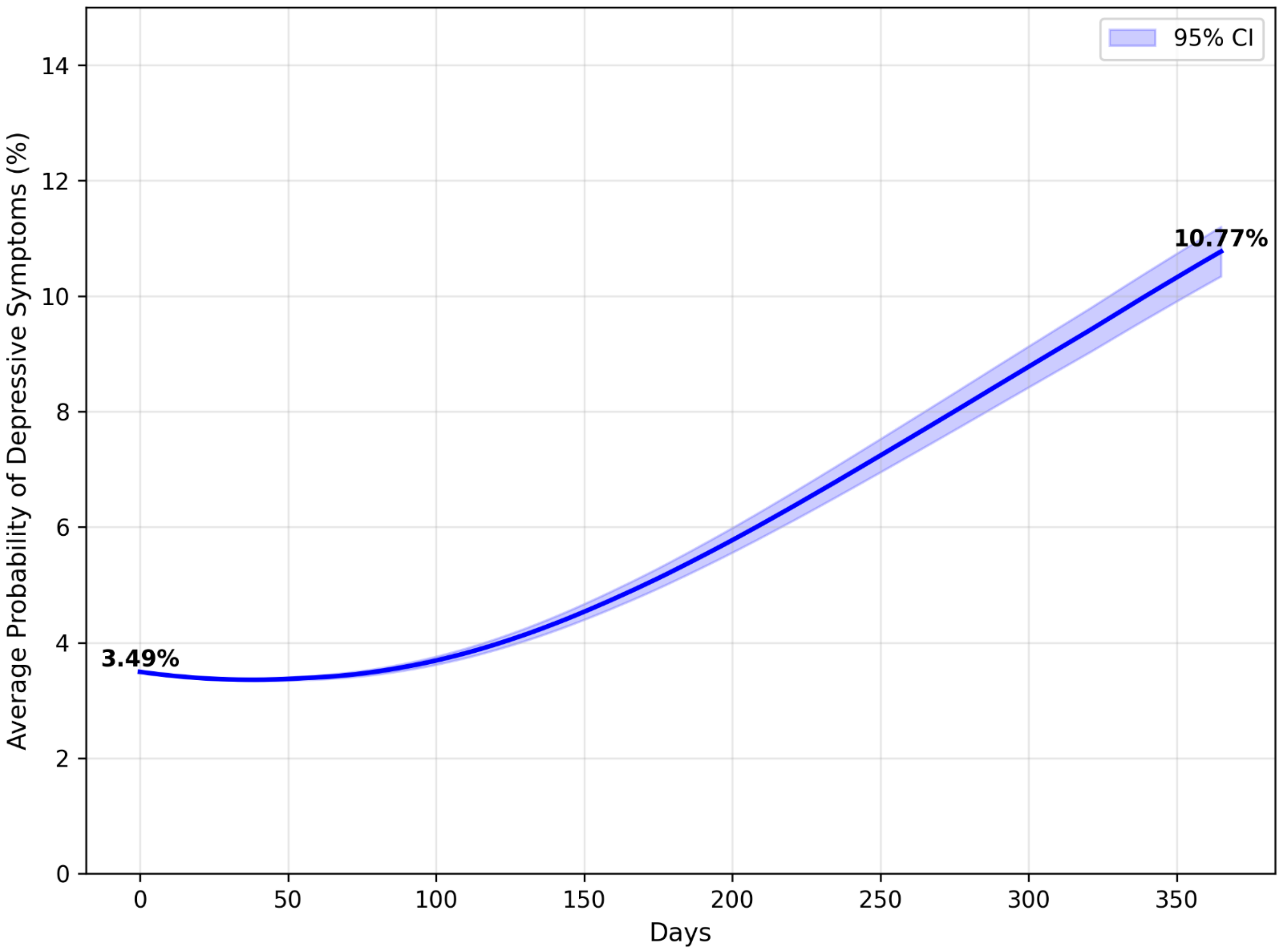

Figure A.7: Probability of developing depressive symptoms in the actual lockdown scenario, 1 February 2021-1 February 2022

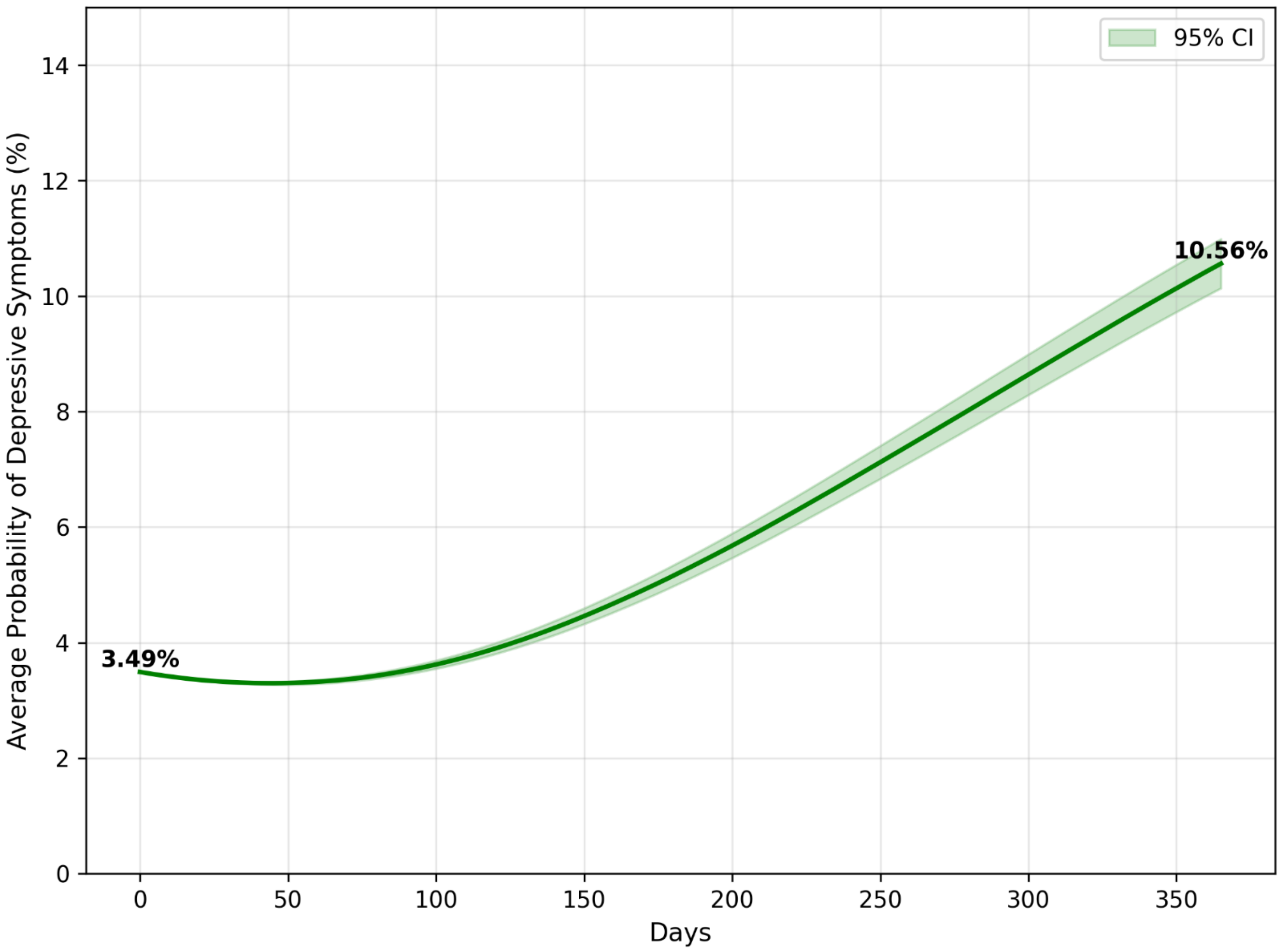

Figure A.8: Probability of developing depressive symptoms in the partial lockdown scenario, 1 February 2021-1 February 2022

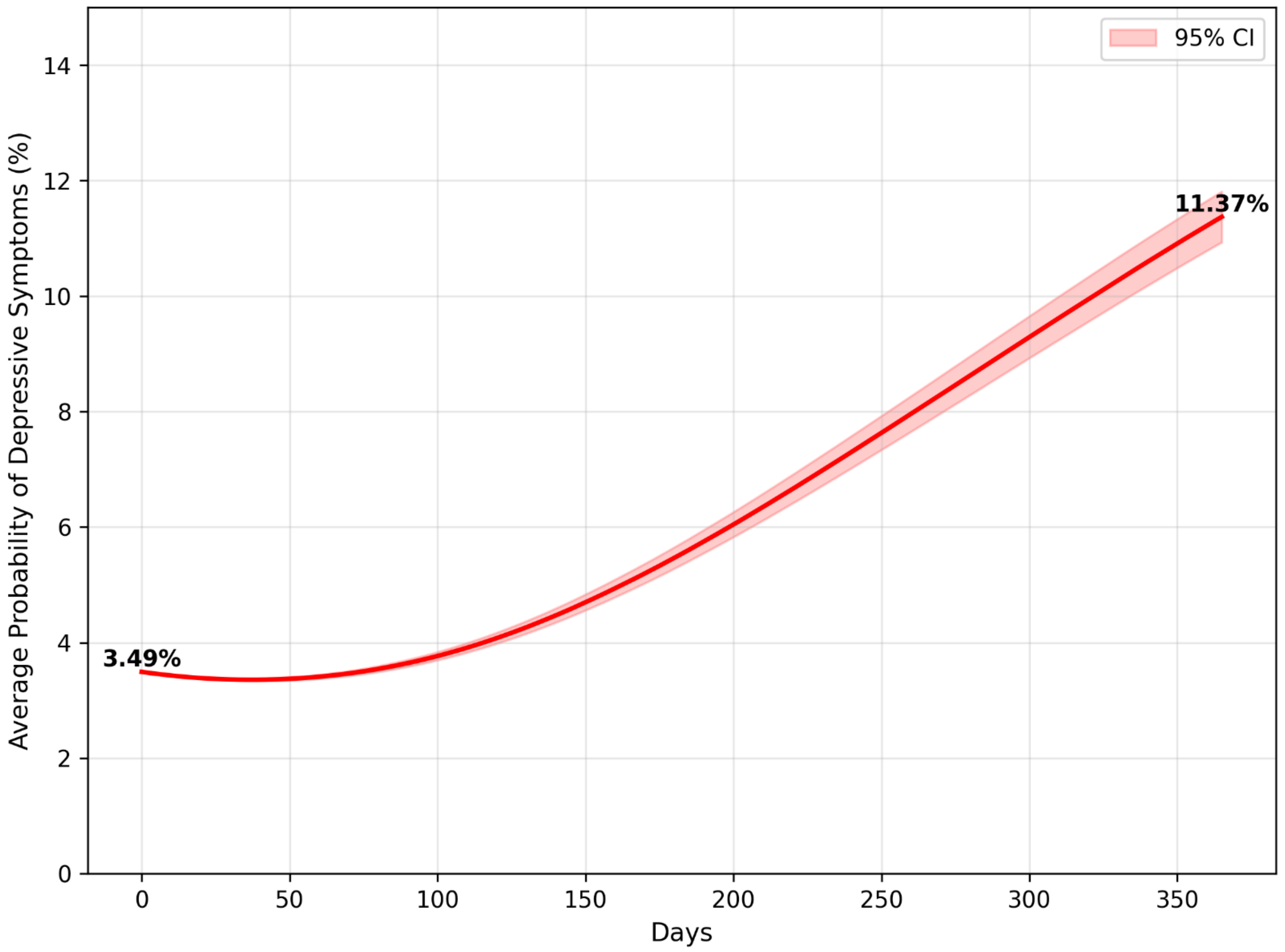

Figure A.9: Probability of developing depressive symptoms in the full lockdown scenario, 1 February 2021-1 February 2022

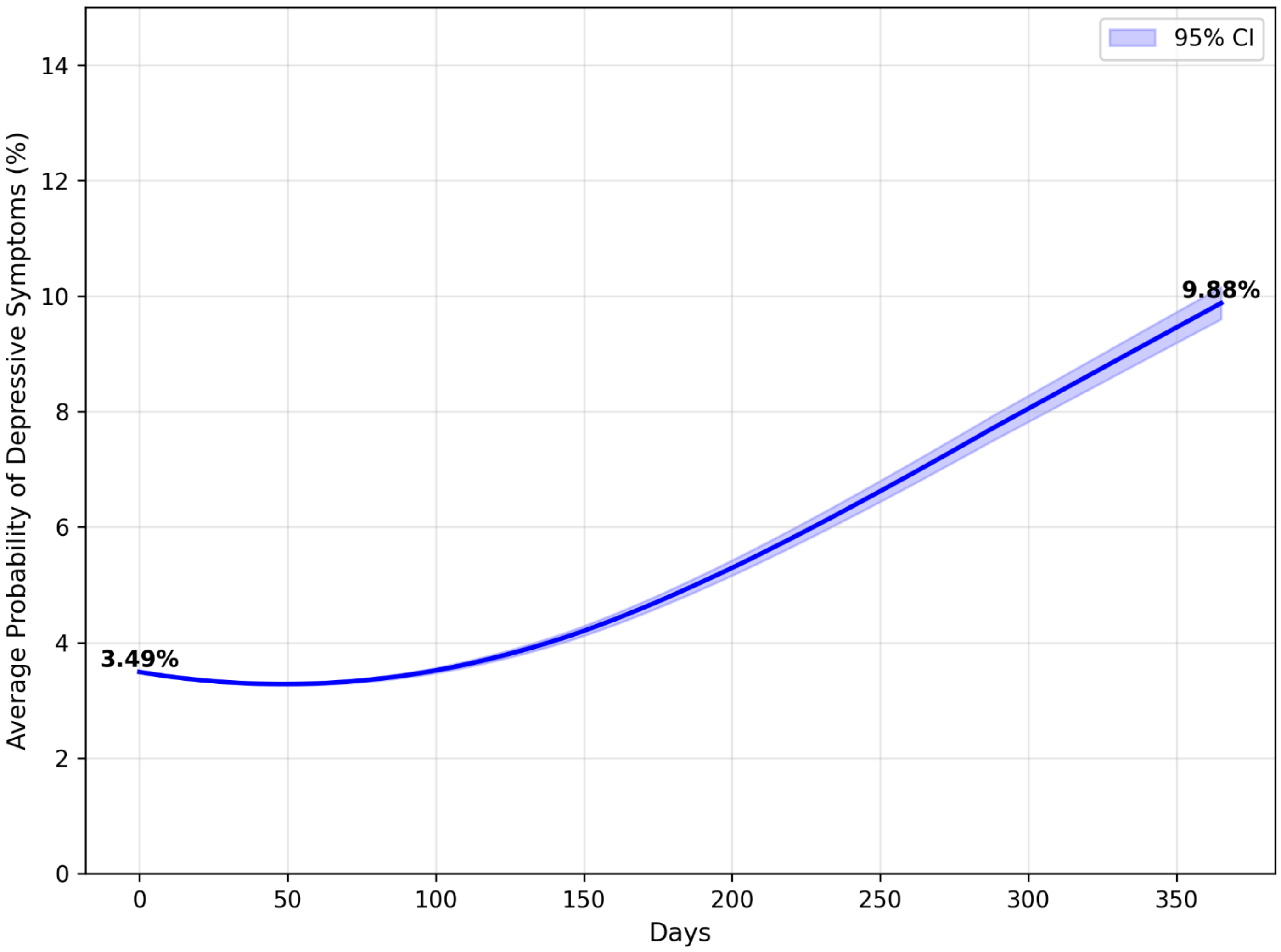

Figure A.10: Probability of developing depressive symptoms in the actual lockdown scenario, 10,000 agents

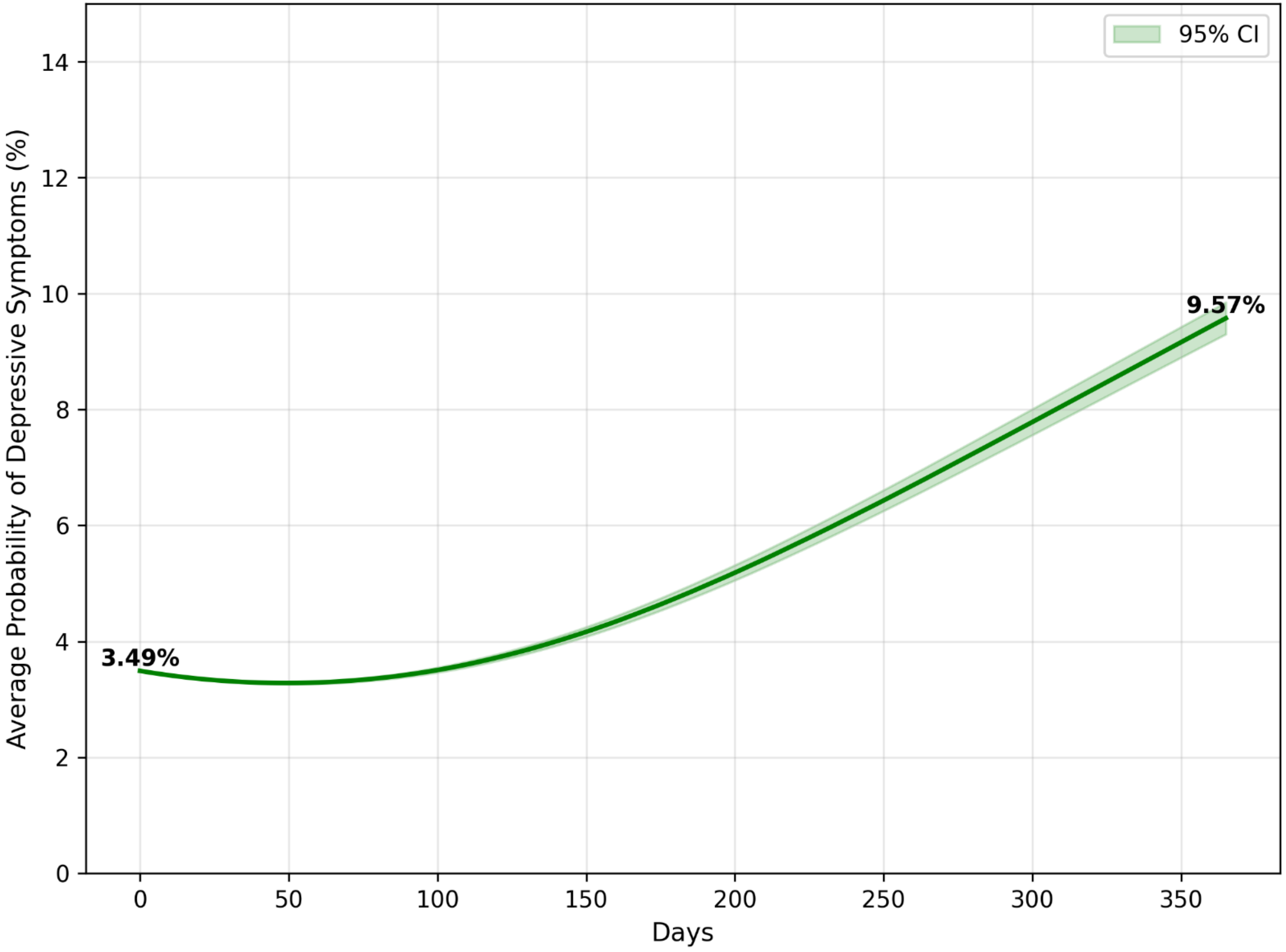

Figure A.11: Probability of developing depressive symptoms in the partial lockdown scenario, 10,000 agents

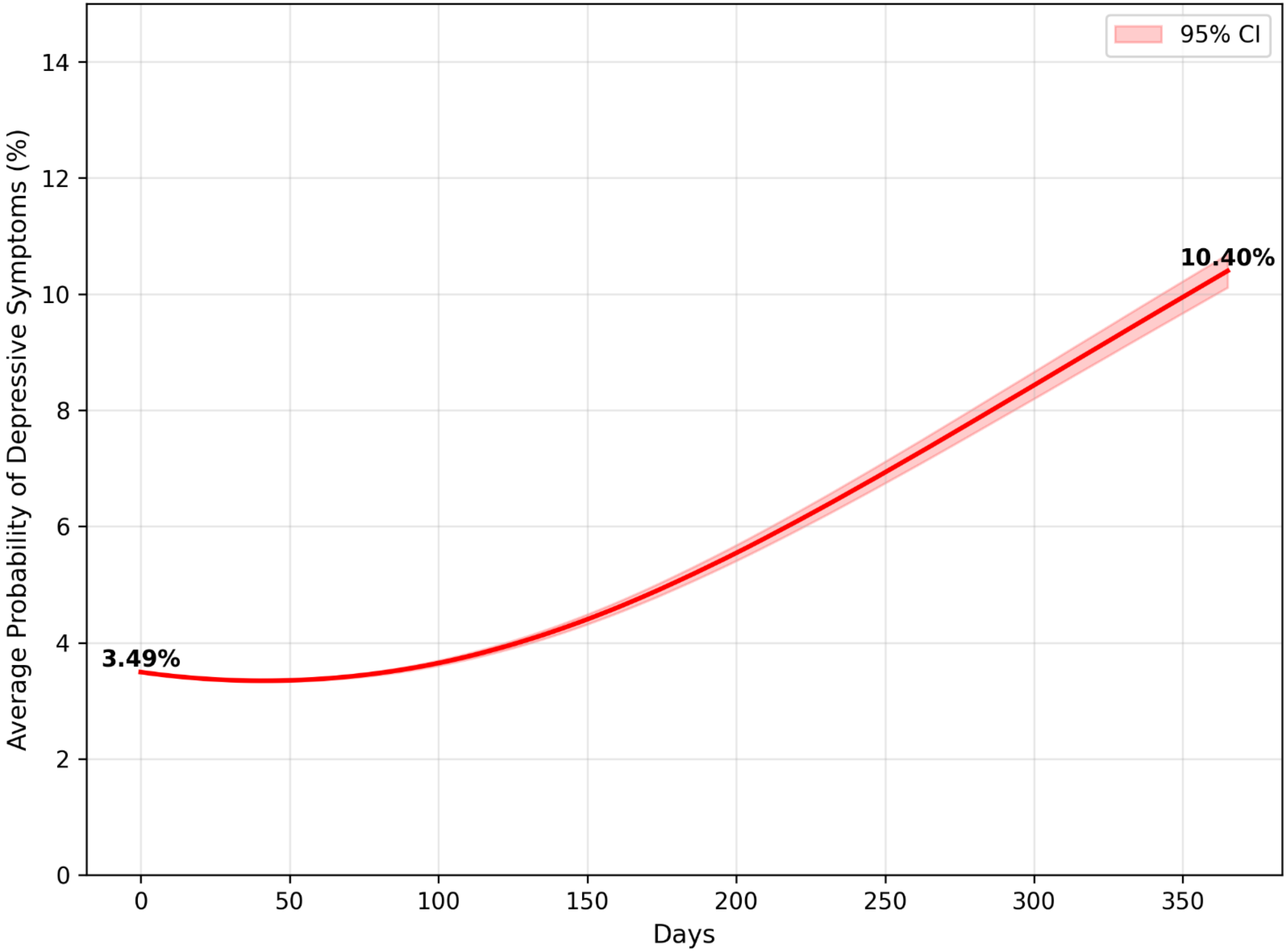

Figure A.12: Probability of developing depressive symptoms in the full lockdown scenario, 10,000 agents
